# Prevalence and characterization of germline RAS pathway variants in children with chronic myeloid leukemia

**DOI:** 10.1038/s41375-026-02952-z

**Published:** 2026-04-24

**Authors:** Tabita Ghete, Laura Gaschler, Manuela Krumbholz, Stephanie Sembill, Yvonne Lisa Behrens, Axel Karow, Matthias Wölfl, Franziska Auer, Julia Hauer, Maria Giulia Carta, Fulvia Ferrazzi, Stephan Hutter, Anselm H. C. Horn, Heinrich Sticht, Paul-Gerhardt Schlegel, Markus Metzler

**Affiliations:** 1 Department of Pediatrics and Adolescent Medicine, University Hospital Erlangen, Erlangen, Germany; 2 Pediatric Oncology Network Bavaria, KIONET, Bavaria, Germany; 3 Bavarian Cancer Research Center (BZKF), Bavaria, Germany; 4 Comprehensive Cancer Center Erlangen-EMN (CCC ER-EMN), Erlangen, Germany; 5 Department of Human Genetics, Hannover Medical School, Hannover, Germany; 6 Institute of Medical Genetics, Carl von Ossietzky University Oldenburg, Oldenburg, Germany; 7 Department of Pediatrics and Adolescent Medicine, University Hospital Würzburg, Würzburg, Germany; 8 Department of Pediatrics, Technical University of Munich, Germany, School of Medicine, Munich, Germany; 9 Institute of Pathology, University Hospital Erlangen, Friedrich-Alexander-Universität Erlangen-Nürnberg, Erlangen, Germany; 10 Department of Nephropathology, Institute of Pathology, University Hospital Erlangen, Friedrich-Alexander-Universität Erlangen-Nürnberg, Erlangen, Germany; 11 MLL Munich Leukemia Laboratory, Munich, Germany; 12 Erlangen National High Performance Computing Center (NHR@FAU), Friedrich-Alexander-Universität Erlangen-Nürnberg, Erlangen, Germany; 13 Bioinformatics, Institute of Biochemistry, Friedrich-Alexander-Universität Erlangen-Nürnberg, Erlangen, Germany

**Keywords:** Chronic myeloid leukaemia, Oncogenesis, Oncogenes, Cancer genetics, Cancer genetics

## Introduction

Genetic predisposition to chronic myeloid leukemia (CML) is not extensively defined, since CML predominantly affects older adults and leukemogenesis is facilitated by age-related accumulation of secondary mutations. Pediatric cases are rare, but studies show that 10% of pediatric patients with CML carry pathogenic germline variants [1], similar to that observed in myelodysplastic syndrome [2].

Moreover, myeloproliferative disorders can be associated with RASopathies [3], but the role of variants within the RAS/RAF/MAPK pathway in CML remains insufficiently understood. The molecular pathogenesis in CML differs from that in myelodysplastic neoplasm or juvenile myelomonocytic leukemia (JMML), because, in CML, *BCR::ABL1* is generally the mandatory, driving oncogene. However, it is not sufficient for the development of CML in all cells [4].

One patient with a pathogenic *SOS1* variant had a clinical diagnosis of Noonan syndrome (NS) and developed CML at an exceptionally young age with a complicated clinical course. This led us to specifically examine the variants within the RAS/RAF/MAPK pathway present in pediatric patients with CML. Through this analysis, germline variants were identified in the genes encoding proteins involved in RAS activation (*SOS1* and *PTPN11*) [5, 6] and in genes encoding negative regulators of RAS (*NF1* and *NF2*) [7, 8].

We hypothesize that germline variants in RAS pathway genes predispose cells to excessive proliferation in combination with the BCR::ABL1 fusion protein, enabling earlier CML onset. To estimate their potential contribution to leukemogenesis pending experimental validation, we classified variants using several in silico tools. Furthermore, we compared them with similar germline variants within the RAS pathway that had previously been reported in individuals with hematological malignancies and/or with RASopathies.

## Material and methods

Whole-exome sequencing and targeted enrichment sequencing were performed on diagnosis and follow-up samples from pediatric patients with *BCR::ABL1*-positive CML from the German pediatric CMLpaed II trial (n = 145; EudraCT 2007-001339-69; Supplementary Methods). All participants or legal guardians provided informed consent in accordance with the Declaration of Helsinki (EK282 122 006, EK 236_18 B). To contextualize the CML variants within the RAS pathway, we compared them with published germline missense variants (“external cohort”) across three groups: hematological malignancies, syndromic hematological malignancies, and syndromic cases (Supplementary Table 1). Overall, 215 variants were identified in *SOS1* (n = 61), *PTPN11* (n = 73), *NF1* (n = 67), and *NF2* (n = 14), distributed among hematological malignancies (n = 19), syndromic hematological malignancies (n = 26), and syndromic cases (n = 171). Germline variants in pediatric patients with CML were characterized according to the American College of Medical Genetics and Genomics (ACMG) classification system [9], and their prevalence was evaluated using the reference population (gnomAD™ version 2.1.1, non-cancer) [10]. Additionally, in silico prediction tools were applied to quantify the potential effect of the germline variant on the protein structure and function (Supplementary Methods).

## Results

Within our cohort, one patient was diagnosed with NS at birth and developed CML in blast phase (CML-BP) at 1.4 years of age (Table 1). Clinical features included short stature, motor delay, and valvular and supravalvular pulmonary stenosis, with a germline *SOS1* R552S variant identified as the underlying genetic cause. No mutations were detected in JMML-associated genes. At presentation, the patient had severe anemia (hemoglobin 6.1 g/dl) and lymphoid blasts in blood. Bone marrow morphology revealed 30–40% lymphoid blasts in a markedly hypocellular marrow. Detection of a *BCR::ABL1* fusion with a major breakpoint and the presence of *BCR::ABL1* in 42% of myeloid interphase nuclei after cytoreductive therapy, confirmed the diagnosis of CML-BP. Treatment included reduced induction chemotherapy (two doses of vincristine and prednisolone) and tyrosine kinase inhibitor treatment. Due to refractory chylothorax, dasatinib was replaced by ponatinib after 15 months. On ponatinib, the patient achieved a major molecular response (*BCR::ABL1/ABL1* ratio ≤1%), but no deeper molecular remission. Allogeneic hematopoietic stem cell transplantation was performed 3.3 years after diagnosis. Two years post-transplantation, the patient remains *BCR::ABL1*-negative despite subsequent graft failure.

**Table 1:**
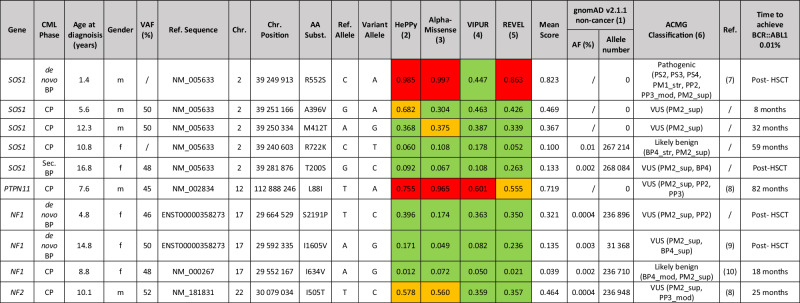
Germline variants in RAS signaling pathway in pediatric patients from CMLpaed II registry (reference genome GRCh37). The reference population was obtained from gnomAD™ v2.1.1 non-cancer (1).

*CML* chronic myeloid leukemia, *BP* blast phase, *CP* chronic phase, *VAF* variant allele frequency, *AF* allele frequency, *m* male, *f* female, *HSCT* hematopoietic stem cell transplantation, *ACMG* American College of Medical Genetics and Genomics.

**Fig. 1:**
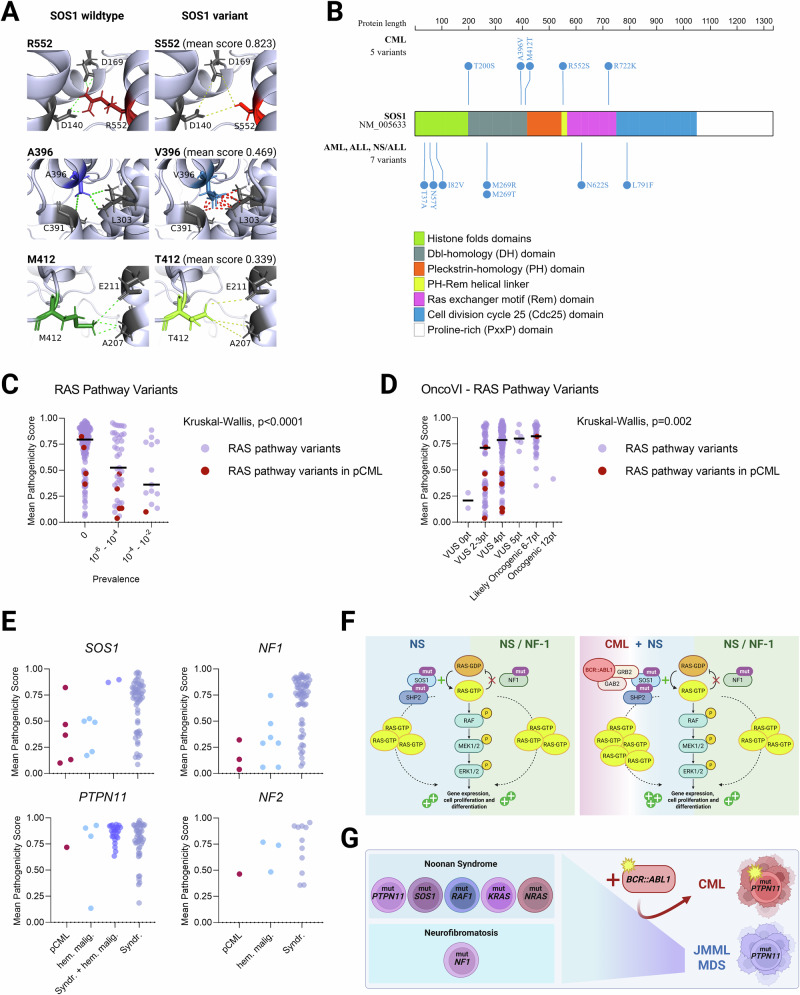
Characteristics of germline variants in RAS pathway genes identified in pediatric patients with chronic myeloid leukemia (pCML). **A** Comparison of structural differences between the SOS1 wildtype and variants encoding the amino acid substitutions in the pCML cohort. Dotted lines represent the distance between amino acids in wildtype SOS1 (green), the modified distance (yellow), or possible clashes (red) in SOS1 variants. Protein structure and amino acid interactions illustrated with PyMOL software. **B** Distribution of germline missense *SOS1* variants in pCML (upper part) and variants in individuals with acute leukemia or Noonan syndrome (NS)-associated leukemia (lower part). **C** Distribution of mean scores of all germline variants (*SOS1, PTPN11, NF1*, and *NF2*) identified in RASopathies and hematological malignancies and their prevalence in the healthy population, as provided by the Genome Aggregation Database (gnomAD™). **D** Distribution of mean scores of all germline variants across the OncoVI categories. The horizontal black lines indicate the median in the respective group. **E** Distribution of mean scores of germline variants across pCML, hematological malignancies (HM), syndromic hematological malignancies (Syndr. HM), and syndromic cases (Syndr.). **F** RAS/RAF/MAPK pathway in NS and neurofibromatosis type 1 (left). In the presence of the constitutively active BCR::ABL1 kinase, hyperactivated RAS leads to an additional increase in downstream processes (right). **G** Germline variants in the RAS signaling pathway are causative of NS and neurofibromatosis type 1. These individuals are predisposed to hematological disorders such as juvenile myelomonocytic leukemia (JMML) and myelodysplastic syndrome (MDS). The additional *BCR::ABL1* translocation may trigger the early manifestation of CML. Created with BioRender.com.

Legend of predicted scores by in silico tools:

The R552S variant resides in a strictly conserved codon and disrupts the autoinhibitory function of the protein through the lack of interaction with the side chains of D140 and D169 in the histone domain (Fig. 1A). *SOS1* R552S was classified as pathogenic by ACMG and in silico predictors (mean score 0.823). In addition, the OncoVI tool for oncogenicity classification [11] labeled the variant as likely oncogenic. R552S belongs to a spectrum of known *SOS1* variants in individuals with NS, with mean scores ranging from 0.086 to 0.969 (median 0.753; Supplementary Table 1). Within the specific context of NS-associated leukemia, R552S scored similarly to previously identified variants in pediatric patients with NS and acquired leukemia (M269R and M269T with a score of 0.898 and 0.871, respectively). While the SOS1 variants in CML are located in similar domains to those in acute leukemia, no cluster between the two cohorts can be defined (Fig. 1B).

Of the 145 pediatric patients for whom genetic analysis was available, 88 had a genetic variant in addition to the *BCR::ABL1* fusion gene. A total of 10 patients (7%; five female and five male) were identified with 10 germline missense variants in four genes associated with the RAS/RAF/MAPK pathway, specifically *SOS1*, *PTPN11*, *NF1*, and *NF2*. The median age at diagnosis was 9.5 (1.4–16.8) years. Germline variants were discovered in six chronic phase CML, three de novo CML-BP, and one secondary CML-BP. According to ACMG guidelines, 7/10 variants were classified as variants of uncertain significance (VUS), 2/10 as likely benign, and 1/10 as pathogenic. Similarly, OncoVI classified 9/10 variants as VUS and 1/10 as likely oncogenic, corresponding to the pathogenic variant classified by ACMG. Their prevalence in the population database (0.0004–0.01%) indicates that they are not frequently occurring single-nucleotide polymorphisms.

When RAS pathway germline variants, both from pediatric patients with CML (n = 10) and those identified in previously published studies (n = 215), are stratified according to their prevalence in the reference population (gnomAD™ version 2.1.1, non-cancer) and their mean score is calculated, we observe that most variants (171/225; 76%) are not present in this database (Fig. 1C). There is a continuum within the CML variants from extremely rare variants predicted to have a more functional impact to the rather benign variants with higher prevalence. OncoVI classified 33/215 variants as likely oncogenic and 1/215 as oncogenic. The median of the predicted mean scores generated by the in silico tools increases with increasing assigned points by OncoVI (Fig. 1D). When we compare the groups separately in each gene, we observe that the mean scores attributed to the CML variants are similar to those of variants identified in hematological malignancies and in syndromic hematological disorders (Fig. 1E and Supplementary Fig. 1). There is significant variation in the median between the analyzed groups for the *SOS1* (*p* = 0.01) and *NF1* (*p* = 0.0006) genes. Variants received varying scores across the four prediction tools. Three in silico tools demonstrate the strongest correlation (*r* ≥ 0.804), whereas one exhibits a moderate correlation with the other tools (Supplementary Fig. 2).

## Discussion

In our study cohort of 145 pediatric patients with CML, 10 carry germline variants in genes associated with the RAS/RAF/MAPK pathway, out of which eight are novel in the context of hematological malignancies. Their presence may contribute to the observed younger median age at CML diagnosis compared to the population in the German Childhood Cancer Registry (9.5 and 13.5 years, respectively). Most variants were classified as VUS. Among the patients with de novo CML-BP, typically occurring in 5% of children with CML [12], three out of four patients carry germline variants.

To our knowledge, this is the first patient reported with NS and CML. This case is distinguished by several particularities, which may relate to the *SOS1* variant in the context of BCR::ABL1 signaling pathways, as this variant results in a gain-of-function and sustained downstream activation [13]. Additionally, the constitutively active tyrosine kinase can bind to the SH2 domain of adapter protein GRB2, which recruits GAB2 and SOS1, ultimately leading to RAS activation and subsequent activation of downstream processes [14]. Germline variants in *PTPN11*, *SOS1*, and *NF1* cause RASopathies, and these individuals are at increased risk for acute leukemia [15] or JMML [3], which could explain the extremely early onset of de novo CML-BP in a 1.4-month-old patient with NS (Fig. 1F). Although *NF2* variants cause neurofibromatosis type 2, they are not known to predispose to hematologic disease, despite the protein’s involvement in the negative regulation of RAS [8].

Although the cohort comprises all patients from the last 10 years in the German CMLpaed registry, including all individuals diagnosed in Germany during this period, the number of patients is still relatively small. Definitive predisposing function of the identified variants has yet to be experimentally proven. Generating appropriate models requires combining these variants with controlled expression of the BCR::ABL1 oncoprotein, which is technically challenging. Therefore, we applied reliable and high-performance in silico tools to classify variants based on predicted structural and functional protein effects. Pathogenic RAS pathway variants are known to predispose to hematologic disorders, as evidenced in the present cohort, wherein the patient with NS carrying the pathogenic *SOS1* R552S variant developed CML-BP at a very young age. Weaker germline variants may not be sufficient for a syndromic phenotype, as observed in nine patients, yet may act as a favorable environment for further genetic alterations (Fig. 1G), thereby increasing the threshold required to initiate leukemogenesis in conjunction with the strong oncogene *BCR::ABL1* and trigger the early manifestation of CML.

## Supplementary information

Supplementary Material

Supplementary Table 1

## Data Availability

For original data, please contact denisia-tabita.ghete@uk-erlangen.de.

## References

[1] Krumbholz M, Dolnik A, Sträng E, Ghete T, Skambraks S, Hutter S, et al. A high proportion of germline variants in pediatric chronic myeloid leukemia. Mol Cancer. 2024;23:206. 39327604 10.1186/s12943-024-02109-5 PMC11426096

[2] Wlodarski MW, Hirabayashi S, Pastor V, Starý J, Hasle H, Masetti R, et al. Prevalence, clinical characteristics, and prognosis of GATA2-related myelodysplastic syndromes in children and adolescents. Blood. 2016;127:1387–97. quiz 518. 26702063 10.1182/blood-2015-09-669937

[3] Kratz CP, Niemeyer CM, Thomas C, Bauhuber S, Matejas V, Bergsträsser E, et al. Mutation analysis of Son of Sevenless in juvenile myelomonocytic leukemia. Leukemia. 2007;21:1108–9. 17315019 10.1038/sj.leu.2404620

[4] Foley SB, Hildenbrand ZL, Soyombo AA, Magee JA, Wu Y, Oravecz-Wilson KI, et al. Expression of BCR/ABL p210 from a knockin allele enhances bone marrow engraftment without inducing neoplasia. Cell Rep. 2013;5:51–60. 24095735 10.1016/j.celrep.2013.08.037 PMC3832111

[5] Sondermann H, Soisson SM, Boykevisch S, Yang S-S, Bar-Sagi D, Kuriyan J. Structural analysis of autoinhibition in the Ras activator Son of Sevenless. Cell. 2004;119:393–405. 15507210 10.1016/j.cell.2004.10.005

[6] Fragale A, Tartaglia M, Wu J, Gelb BD. Noonan syndrome-associated SHP2/PTPN11 mutants cause EGF-dependent prolonged GAB1 binding and sustained ERK2/MAPK1 activation. Hum Mutat. 2004;23:267–77. 14974085 10.1002/humu.20005

[7] Yunoue S, Tokuo H, Fukunaga K, Feng L, Ozawa T, Nishi T, et al. Neurofibromatosis type I tumor suppressor neurofibromin regulates neuronal differentiation via its GTPase-activating protein function toward Ras. J Biol Chem. 2003;278:26958–69. 12730209 10.1074/jbc.M209413200

[8] Cui Y, Groth S, Troutman S, Carlstedt A, Sperka T, Riecken LB, et al. The NF2 tumor suppressor merlin interacts with Ras and RasGAP, which may modulate Ras signaling. Oncogene. 2019;38:6370–81. 31312020 10.1038/s41388-019-0883-6 PMC6756068

[9] Richards S, Aziz N, Bale S, Bick D, Das S, Gastier-Foster J, et al. Standards and guidelines for the interpretation of sequence variants: a joint consensus recommendation of the American College of Medical Genetics and Genomics and the Association for Molecular Pathology. Genet Med. 2015;17:405–24. 25741868 10.1038/gim.2015.30 PMC4544753

[10] Karczewski KJ, Francioli LC, Tiao G, Cummings BB, Alföldi J, Wang Q, et al. The mutational constraint spectrum quantified from variation in 141,456 humans. Nature. 2020;581:434–43. 32461654 10.1038/s41586-020-2308-7 PMC7334197

[11] Carta MG, Tögel L, Hölsken A, Schubart C, Sticht H, Stöhr R, et al. Oncogenicity Variant Interpreter (OncoVI): oncogenicity guidelines implementation to support somatic variants interpretation in precision oncology. J Mol Diagn. 2026:S1525–1578(26)00054–1. 10.1016/j.jmoldx.2026.03.004 PMC13269341 41936821

[12] Sembill S, Göhring G, Schirmer E, Lutterloh F, Suttorp M, Metzler M, et al. Paediatric chronic myeloid leukaemia presenting in de novo or secondary blast phase - a comparison of clinical and genetic characteristics. Br J Haematol. 2021;193:613–8. 33690887 10.1111/bjh.17378

[13] Roberts AE, Araki T, Swanson KD, Montgomery KT, Schiripo TA, Joshi VA, et al. Germline gain-of-function mutations in SOS1 cause Noonan syndrome. Nature Genetics. 2007;39:70–4. 17143285 10.1038/ng1926

[14] Sattler M, Mohi MG, Pride YB, Quinnan LR, Malouf NA, Podar K, et al. Critical role for Gab2 in transformation by BCR/ABL. Cancer Cell. 2002;1:479–92. 12124177 10.1016/s1535-6108(02)00074-0

[15] Cavé H, Caye A, Strullu M, Aladjidi N, Vignal C, Ferster A, et al. Acute lymphoblastic leukemia in the context of RASopathies. Eur J Med Genet. 2016;59:173–8. 26855057 10.1016/j.ejmg.2016.01.003

