# Supplementary material for: Prevalence and characterization of germline RAS pathway variants in children with chronic myeloid leukemia
Source: Leukemia. 2026 Apr 24;40(7):1527–31. doi: 10.1038/s41375-026-02952-z (PMC13322959; doi:10.1038/s41375-026-02952-z)
Supplement: Supplementary file 1 — Supplementary Material [file 41375_2026_2952_MOESM1_ESM.docx]

## Supplementary Methods

### Pediatric CML study cohort and genetic analyses

DNA was isolated from frozen blood and/or bone marrow samples at diagnosis or in remission (*BCR::ABL1* <5%) using the QIAamp DNA Blood Mini Kit (Qiagen, Hilden, Germany) or AllPrep DNA/RNA Kit (Qiagen) according to the manufacturer’s instructions. For validation of germline variants, fingernail or saliva samples were analyzed by Sanger sequencing in addition to remission samples, if available. For whole-exome sequencing, the libraries were sequenced on HiSeq 2000 with 100 bp paired-end reads using a 200-cycle TruSeq SBS v3 kit (reference genome GRCh37), and the filtering was performed on the output of VarScan2 v 2.3.9 (1) after annotation using Annovar (2) release 20150322. For the targeted enrichment sequencing, a custom amplicon panel with 62 target genes (3) or 148 leukemia-associated genes (4) was designed. This included all genes for which germline variants have been identified with whole-exome sequencing, and genes commonly mutated in CML and other hematologic malignancies. Data analysis was performed only on variants at tumor allele frequency >5% and that are rarely present in healthy individuals (<1% in the database SNP134) (5).

### External cohort

The following terms and their combinations were used to search relevant publications on PubMed: "*SOS1*," "*PTPN11*," "*NF1*," "*NF2*," "Noonan syndrome," "RASopathies," "acute leukemia," "chronic leukemia," "juvenile myelomonocytic leukemia," "myelodysplastic syndrome," and "neurofibromatosis." An overview of the 215 germline variants and the corresponding studies is provided in Supplementary Table 1.

### *In silico* classification of genetic variants

We applied *in silico* prediction tools to quantify the potential effect of the germline variant on the protein structure and function. For this purpose, four algorithms were employed. AlphaMissense is one of the leading algorithms based on correlation with functional impact (6). VIPUR integrates thermodynamic simulations and predicts the loss of molecular function (7). As a more specialized tool, the Hematological Predictor of Pathogenicity (HePPy) combines 10 *in silico* predictor scores and four phylogenetic conservation scores and was trained on hematopathological somatic missense variants (8). Similarly, the Rare Exome Variant Ensemble Learner (REVEL) incorporates 13 individual tools (9). The scores were categorized into classes as follows: AlphaMissense: >0.564, likely pathogenic; 0.340-0.564, ambiguous; ≤0.339, likely benign. VIPUR: >0.5, deleterious; <0.5, neutral. HePPy and REVEL: >0.75, probably pathogenic; 0.5-0.75, possibly pathogenic; <0.5, probably benign. To allow for an easier comparison of different variants, a mean pathogenicity score from all four *in silico* methods was calculated. This score is referred to as the 'mean score'. For further assessment, we evaluated our variants with the Oncogenicity Variant Interpreter (OncoVI), a tool for variant oncogenicity classification (10). The PyMOL software was used to construct protein models that illustrate the effects of the amino acid substitution (11). Lollipop plots showing the variant distribution across protein domains were designed with ProteinPaint (12). Statistical and correlation analyses were conducted using GraphPad prism software (v9.5.1) (GraphPad Software, San Diego, California, USA, www.graphpad.com (2023)). To compare the pathogenicity scores between the analysed groups, the Kruskal-Wallis test was used (p values of <0.05 were considered statistically significant).

## Supplementary Figures


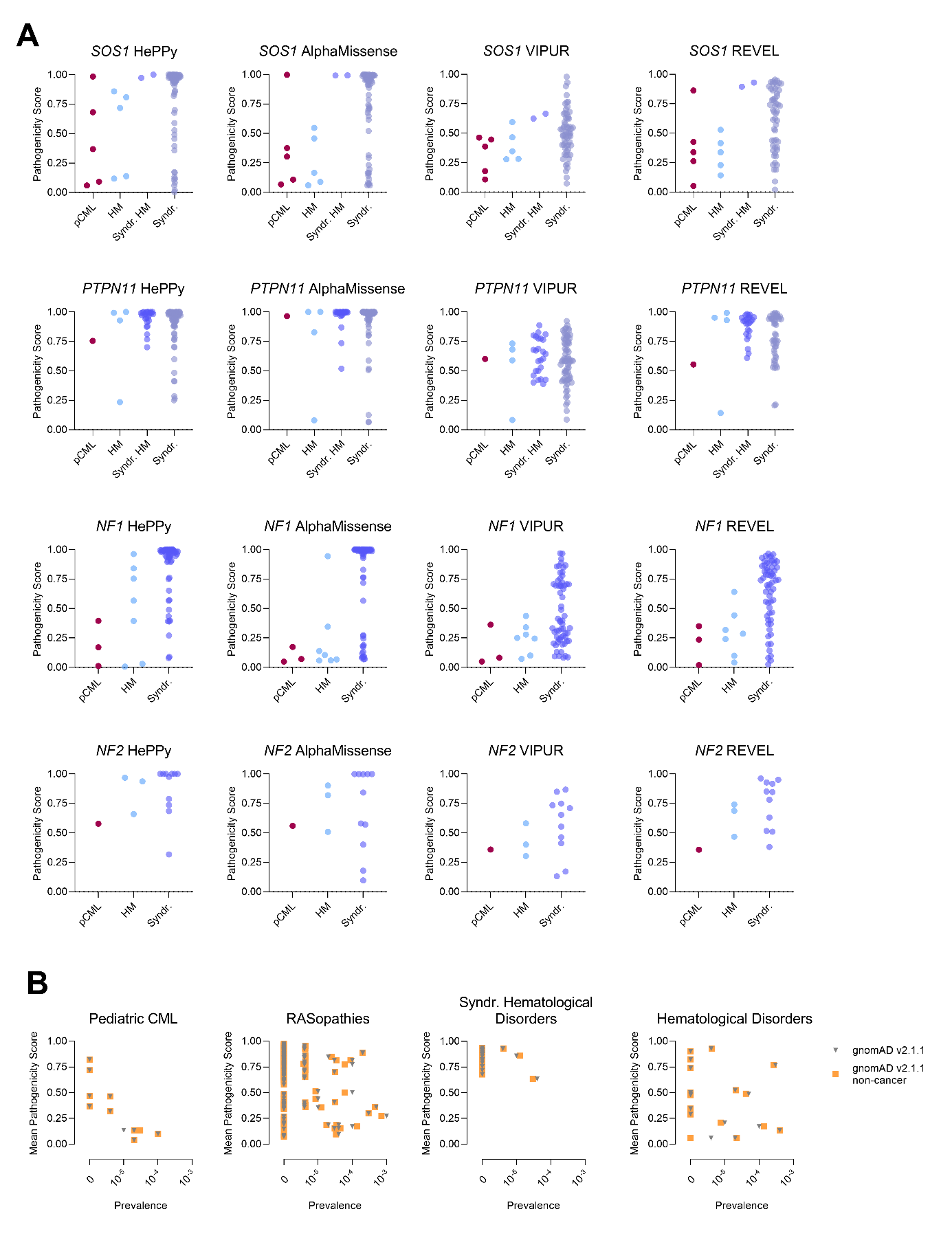


Supplementary Figure 1. (A) Distribution of mean scores of *SOS1*, *PTPN11*, *NF1*, and *NF2* germline variants across pediatric chronic myeloid leukemia (pCML), hematological malignancies (HM), syndromic hematological malignancies (Syndr. HM), and syndromic cases (Syndr.) as classified by each individual predictor. (B) Distribution of mean scores of these variants across their prevalence in the reference population gnomAD™ v2.1.1 and gnomAD™ v2.1.1 non-cancer.


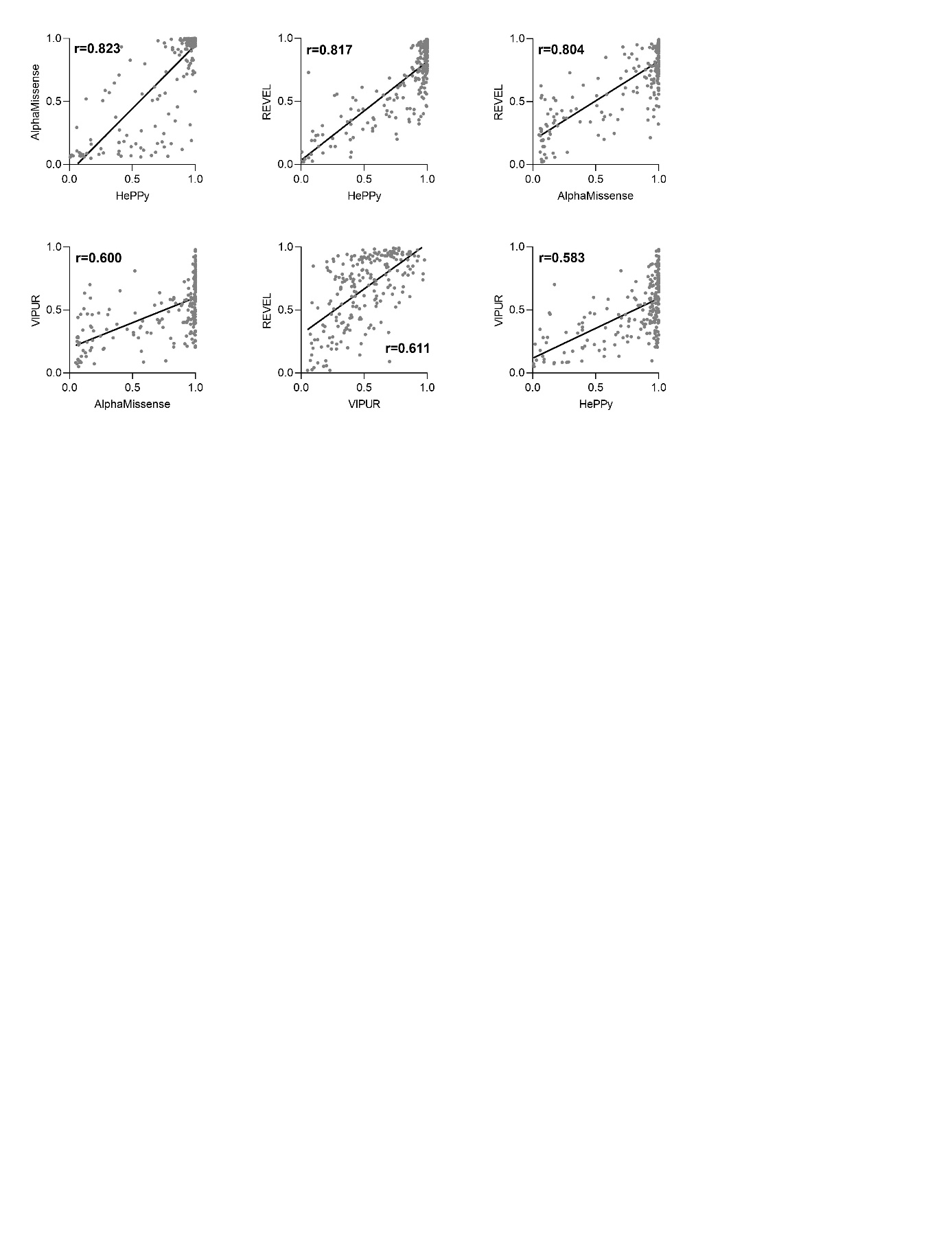


Supplementary Figure 2. Pearson correlation analysis of the germline variants’ score (*SOS1*, *PTPN11*, *NF1*, and *NF2*) between four in silico tools: HePPy, AlphaMissense, REVEL, and VIPUR.

## References

1. Koboldt DC, Zhang Q, Larson DE, Shen D, McLellan MD, Lin L, et al. VarScan 2: somatic mutation and copy number alteration discovery in cancer by exome sequencing. Genome Res. 2012;22(3):568-76.

2. Wang K, Li M, Hakonarson H. ANNOVAR: functional annotation of genetic variants from high-throughput sequencing data. Nucleic Acids Res. 2010;38(16):e164.

3. Krumbholz M, Dolnik A, Sträng E, Ghete T, Skambraks S, Hutter S, et al. A high proportion of germline variants in pediatric chronic myeloid leukemia. Mol Cancer. 2024;23(1):206.

4. Behrens YL, Gaschler L, Nienhold R, Reinkens T, Schirmer E, Knöß S, et al. Somatic variant profiling in chronic phase pediatric chronic myeloid leukemia. Haematologica. 2024;109(3):942-7.

5. Sherry ST, Ward MH, Kholodov M, Baker J, Phan L, Smigielski EM, et al. dbSNP: the NCBI database of genetic variation. Nucleic Acids Res. 2001;29(1):308-11.

6. Cheng J, Novati G, Pan J, Bycroft C, Žemgulytė A, Applebaum T, et al. Accurate proteome-wide missense variant effect prediction with AlphaMissense. Science. 2023;381(6664):eadg7492.

7. Baugh EH, Simmons-Edler R, Müller CL, Alford RF, Volfovsky N, Lash AE, et al. Robust classification of protein variation using structural modelling and large-scale data integration. Nucleic Acids Res. 2016;44(6):2501-13.

8. Hutter S, Baer C, Walter W, Kern W, Haferlach C, Haferlach T. A Novel Machine Learning Based in silico Pathogenicity Predictor for Missense Variants in a Hematological Setting. Blood. 2019;134(Supplement_1):2090-.

9. Ioannidis NM, Rothstein JH, Pejaver V, Middha S, McDonnell SK, Baheti S, et al. REVEL: An Ensemble Method for Predicting the Pathogenicity of Rare Missense Variants. The American Journal of Human Genetics. 2016;99(4):877-85.

10. Carta MG, Tögel L, Hölsken A, Schubart C, Sticht H, Stöhr R, et al. Oncogenicity Variant Interpreter (OncoVI): oncogenicity guidelines implementation to support somatic variants interpretation in precision oncology. The Journal of Molecular Diagnostics. 2026.

11. Schrödinger LaWD. PyMOL. 2.5.7 ed2020.

12. Zhou X, Edmonson MN, Wilkinson MR, Patel A, Wu G, Liu Y, et al. Exploring genomic alteration in pediatric cancer using ProteinPaint. Nat Genet. 2016;48(1):4-6.
