# Supplementary material for: Prevalence and characterization of germline RAS pathway variants in children with chronic myeloid leukemia
Source: Leukemia. 2026 Apr 24;40(7):1527–31. doi: 10.1038/s41375-026-02952-z (PMC13322959; doi:10.1038/s41375-026-02952-z)
Supplement: Supplementary file 2 — Supplementary Table 1 [file 41375_2026_2952_MOESM2_ESM.docx]

Supplementary Table 1. Germline variants in RAS signaling pathway in patients with hematological disorders, in patients with RASopathies and acquired hematological disorders, and patients with RASopathies (reference genome GRCh37). The reference population was obtained from gnomAD™ v2.1.1 non-cancer (1).

| **Gene** | **Malignancy** | **Ref. Sequence** | **Chr.** | **Chr. Position** | **AA Substitution** | **Ref. Allele** | **Variant Allele** | **HePPy**  **(2)** | **Alpha-Missense**  **(3)** | **VIPUR**  **(4)** | **REVEL**  **(5)** | **Mean Score** | **gnomAD** **v2.1.1 non-cancer (1)** | | **OncoVI Classification (6)** | | | **Ref.** |
| --- | --- | --- | --- | --- | --- | --- | --- | --- | --- | --- | --- | --- | --- | --- | --- | --- | --- | --- |
|  |  |  |  |  |  |  |  |  |  |  |  |  | **AF (%)** | **Allele number** | **Points** | **Class** | **Criteria** |  |
| *SOS1* | NS | NM_005633 | 2 | 39 250 247 | C441Y | C | T | 1.000 | 0.999 | 0.979 | 0.898 | 0.969 | / | 0 | 4 | VUS | OM1, OP1, OP4 | (7) |
| *SOS1* | NS | NM_005633 | 2 | 39 250 269 | G434R | C | G | 0.982 | 1.000 | 0.931 | 0.927 | 0.960 | / | 0 | 6 | LO | OS1, OP1, OP4 | (8) |
| *SOS1* | NS | NM_005633 | 2 | 39 250 275 | W432R | A | G | 1.000 | 1.000 | 0.900 | 0.931 | 0.958 | / | 0 | 4 | VUS | OM1, OP1, OP4 | (7) |
| *SOS1* | NS | NM_005633 | 2 | 39 249 920 | L550P | A | G | 0.998 | 0.993 | 0.763 | 0.947 | 0.925 | / | 0 | 4 | VUS | OM1, OP1, OP4 | (7) |
| *SOS1* | NS | NM_005633 | 2 | 39 285 906 | W85R | A | T | 0.999 | 1.000 | 0.824 | 0.794 | 0.904 | / | 0 | 4 | VUS | OM1, OP1, OP4 | (9) |
| *SOS1* | NS/ALL | NM_005633 | 2 | 39 278 343 | M269R | A | C | 1.000 | 0.993 | 0.666 | 0.931 | 0.898 | / | 0 | 6 | LO | OS1, OP1, OP4 | (10) |
| *SOS1* | NS | NM_005633 | 2 | 39 285 926 | F78C | A | C | 1.000 | 0.998 | 0.681 | 0.865 | 0.886 | 0.02 | 267 508 | 4 | VUS | OM1, OP1, OP4 | (11) |
| *SOS1* | NS | NM_005633 | 2 | 39 250 268 | G434K | CC | TT | / | 1.000 | 0.766 | / | 0.883 | / | 0 | 3 | VUS | OM1, OP4 | (12) |
| *SOS1* | NS/ALL | NM_005633 | 2 | 39 278 343 | M269T | A | G | 0.973 | 0.992 | 0.624 | 0.895 | 0.871 | / | 0 | 4 | VUS | OM1, OP1, OP4 | (10) |
| *SOS1* | NS | NM_005633 | 2 | 39 249 914 | R552T | C | G | 0.996 | 0.995 | 0.507 | 0.955 | 0.863 | / | 0 | 4 | VUS | OM1, OP1, OP4 | (13) |
| *SOS1* | NS | NM_005633 | 2 | 39 250 100 | L490R | A | C | 1.000 | 0.961 | 0.547 | 0.939 | 0.862 | / | 0 | 4 | VUS | OM1, OP1, OP4 | (12) |
| *SOS1* | NS | NM_005633 | 2 | 39 249 914 | R552M | C | A | 0.996 | 0.994 | 0.545 | 0.903 | 0.860 | 0.0004 | 236 374 | 4 | VUS | OM1, OP1, OP4 | (12) |
| *SOS1* | NS | NM_005633 | 2 | 39 262 417 | Y337C | T | C | 1.000 | 0.945 | 0.672 | 0.780 | 0.849 | 0.003 | 236 748 | 4 | VUS | OM1, OP1, OP4 | (8) |
| *SOS1* | NS | NM_005633 | 2 | 39 249 915 | R552G | T | C | 0.985 | 0.980 | 0.477 | 0.921 | 0.841 | 0.0004 | 236 388 | 6 | LO | OS1, OP1, OP4 | (8) |
| *SOS1* | NS | NM_005633 | 2 | 39 250 259 | I437T | A | G | 0.949 | 0.954 | 0.533 | 0.922 | 0.840 | / | 0 | 4 | VUS | OM1, OP1, OP4 | (12) |
| *SOS1* | NS | NM_005633 | 2 | 39 241 979 | F623I | A | T | 0.998 | 0.988 | 0.593 | 0.733 | 0.828 | / | 0 | 4 | VUS | OM1, OP1, OP4 | (11) |
| *SOS1* | NS; pCML | NM_005633 | 2 | 39 249 913 | R552S | C | A | 0.985 | 0.997 | 0.447 | 0.863 | 0.823 | / | 0 | 6 | LO | OS1, OP1, OP4 | (7) |
| *SOS1* | NS | NM_005633 | 2 | 39 278 394 | I252T | A | G | 0.979 | 0.716 | 0.637 | 0.935 | 0.817 | 0.009 | 236 776 | 4 | VUS | OM1, OP1, OP4 | (12) |
| *SOS1* | NS | NM_005633 | 2 | 39 249 915 | R552W | T | A | 0.986 | 0.971 | 0.484 | 0.816 | 0.814 | / | 0 | 4 | VUS | OM1, OP1, OP4 | (9) |
| *SOS1* | NS | NM_005633 | 2 | 39 239 471 | W729L | C | A | 0.971 | 0.986 | 0.750 | 0.524 | 0.808 | / | 0 | 4 | VUS | OM1, OP1, OP4 | (7) |
| *SOS1* | NS | NM_005633 | 2 | 39 250 272 | E433K | C | T | 0.976 | 0.982 | 0.459 | 0.778 | 0.799 | 0.0004 | 236 228 | 4 | VUS | OM1, OP1, OP4 | (7) |
| *SOS1* | NS | NM_005633 | 2 | 39 285 854 | P102R | G | C | 0.995 | 0.729 | 0.556 | 0.877 | 0.789 | / | 0 | 4 | VUS | OM1, OP1, OP4 | (14) |
| *SOS1* | NS | NM_005633 | 2 | 39 239 460 | I733F | T | A | 0.948 | 0.963 | 0.630 | 0.571 | 0.778 | / | 0 | 4 | VUS | OM1, OP1, OP4 | (7) |
| *SOS1* | NS | NM_005633 | 2 | 39 283 845 | K170E | T | C | 0.952 | 0.787 | 0.487 | 0.879 | 0.776 | / | 0 | 4 | VUS | OM1, OP1, OP4 | (14) |
| *SOS1* | NS | NM_005633 | 2 | 39 250 079 | R497Q | C | T | 0.999 | 0.939 | 0.417 | 0.744 | 0.775 | 0.006 | 267 346 | 4 | VUS | OM1, OP1, OP4 | (15) |
| *SOS1* | NS | NM_005633 | 2 | 39 250 299 | E424K | C | T | 0.971 | 0.931 | 0.401 | 0.788 | 0.773 | 0.0004 | 236 058 | 4 | VUS | OM1, OP1, OP4 | (12) |
| *SOS1* | NS | NM_005633 | 2 | 39 250 305 | M422V | T | C | 0.819 | 0.912 | 0.725 | 0.625 | 0.770 | / | 0 | 4 | VUS | OM1, OP1, OP4 | (12) |
| *SOS1* | NS | NM_005633 | 2 | 39 249 927 | S548R | T | G | 0.932 | 0.987 | 0.394 | 0.750 | 0.766 | / | 0 | 4 | VUS | OM1, OP1, OP4 | (8) |
| *SOS1* | NS | NM_005633 | 2 | 39 250 125 | G482R | C | G | 0.956 | 0.941 | 0.499 | 0.614 | 0.753 | / | 0 | 4 | VUS | OM1, OP1, OP4 | (12) |
| *SOS1* | NS | NM_005633 | 2 | 39 239 474 | K728I | T | A | 0.950 | 0.995 | 0.401 | 0.608 | 0.739 | / | 0 | 4 | VUS | OM1, OP1, OP4 | (16) |
| *SOS1* | NS | NM_005633 | 2 | 39 278 352 | T266K | G | T | 0.837 | 0.888 | 0.535 | 0.696 | 0.739 | / | 0 | 4 | VUS | OM1, OP1, OP4 | (8) |
| *SOS1* | NS | NM_005633 | 2 | 39 285 837 | E108K | C | T | 0.971 | 0.957 | 0.313 | 0.668 | 0.727 | / | 0 | 4 | VUS | OM1, OP1, OP4 | (7) |
| *SOS1* | NS | NM_005633 | 2 | 39 240 664 | Y702H | A | G | 0.930 | 0.962 | 0.315 | 0.681 | 0.722 | 0.0004 | 236 390 | 4 | VUS | OM1, OP1, OP4 | (7) |
| *SOS1* | NS | NM_005633 | 2 | 39 224 428 | Q977R | T | C | 0.950 | 0.960 | 0.552 | 0.380 | 0.711 | / | 0 | 4 | VUS | OM1, OP1, OP4 | (7) |
| *SOS1* | NS | NM_005633 | 2 | 39 249 914 | R552K | C | T | 0.973 | 0.827 | 0.206 | 0.837 | 0.711 | / | 0 | 4 | VUS | OM1, OP1, OP4 | (7) |
| *SOS1* | NS | NM_005633 | 2 | 39 239 306 | I784T | A | G | 0.982 | 0.742 | 0.362 | 0.742 | 0.707 | 0.0004 | 236 500 | 4 | VUS | OM1, OP1, OP4 | (12) |
| *SOS1* | NS | NM_005633 | 2 | 39 285 824 | P112R | G | C | 0.934 | 0.915 | 0.578 | 0.353 | 0.695 | / | 0 | 4 | VUS | OM1, OP1, OP4 | (12) |
| *SOS1* | NS | NM_005633 | 2 | 39 249 923 | T549K | G | T | 0.908 | 0.699 | 0.424 | 0.685 | 0.679 | / | 0 | 4 | VUS | OM1, OP1, OP4 | (12) |
| *SOS1* | NS | NM_005633 | 2 | 39 234 309 | E846K | C | T | 0.864 | 0.914 | 0.403 | 0.443 | 0.656 | / | 0 | 6 | LO | OS1, OP1, OP4 | (8) |
| *SOS1* | NS | NM_005633 | 2 | 39 233 663 | P894R | G | C | 0.855 | 0.675 | 0.539 | 0.357 | 0.607 | / | 0 | 4 | VUS | OM1, OP1, OP4 | (12) |
| *SOS1* | NS | NM_005633 | 2 | 39 262 581 | D309Y | C | A | 0.959 | 0.315 | 0.505 | 0.534 | 0.578 | / | 0 | 4 | VUS | OM1, OP1, OP4 | (8) |
| *SOS1* | ALL | NM_005633 | 2 | 39 294 813 | N57Y | T | A | 0.809 | 0.166 | 0.595 | 0.529 | 0.525 | 0.002 | 236 860 | 4 | VUS | OM1, OP1, OP4 | (17) |
| *SOS1* | NS | NM_005633 | 2 | 39 234 258 | Q863K | G | T | 0.676 | 0.616 | 0.322 | 0.396 | 0.503 | 0.006 | 267 998 | 4 | VUS | OM1, OP1, OP4 | (18) |
| *SOS1* | AML | NM_005633 | 2 | 39 239 286 | L791F | G | A | 0.859 | 0.457 | 0.347 | 0.338 | 0.500 | / | 0 | 4 | VUS | OM1, OP1, OP4 | (17) |
| *SOS1* | ALL | NM_005633 | 2 | 39 285 915 | I82V | T | C | 0.718 | 0.547 | 0.279 | 0.416 | 0.490 | 0.004 | 267 350 | 4 | VUS | OM1, OP1, OP4 | (17) |
| *SOS1* | NS | NM_005633 | 2 | 39 250 136 | P478R | G | C | 0.395 | 0.710 | 0.398 | 0.439 | 0.486 | / | 0 | 4 | VUS | OM1, OP1, OP4 | (11) |
| *SOS1* | pCML | NM_005633 | 2 | 39 251 166 | A396V | G | A | 0.682 | 0.304 | 0.463 | 0.426 | 0.469 | / | 0 | 4 | VUS | OM1, OP1, OP4 | / |
| *SOS1* | NS | NM_005633 | 2 | 39 281 814 | L221V | G | C | 0.700 | 0.167 | 0.372 | 0.380 | 0.405 | / | 0 | 4 | VUS | OM1, OP1, OP4 | (18) |
| *SOS1* | NS | NM_005633 | 2 | 39 250 138 | Q477H | C | A | 0.456 | 0.231 | 0.476 | 0.428 | 0.398 | / | 0 | 2 | VUS | OM1, OP4, SBP1 | (11) |
| *SOS1* | NS | NM_005633 | 2 | 39 251 221 | T378A | T | C | 0.357 | 0.647 | 0.316 | 0.247 | 0.392 | / | 0 | 4 | VUS | OM1, OP1, OP4 | (14) |
| *SOS1* | NS | NM_005633 | 2 | 39 250 136 | P478L | G | A | 0.491 | 0.141 | 0.469 | 0.439 | 0.385 | / | 0 | 4 | VUS | OM1, OP1, OP4 | (11) |
| *SOS1* | NS | NM_005633 | 2 | 39 213 008 | H1320R* | T | C | 0.589 | 0.114 | 0.510 | 0.306 | 0.380 | / | 0 | 2 | VUS | OP1, OP4 | (7) |
| *SOS1* | pCML | NM_005633 | 2 | 39 250 334 | M412T | A | G | 0.368 | 0.375 | 0.387 | 0.339 | 0.367 | / | 0 | 4 | VUS | OM1, OP1, OP4 | / |
| *SOS1* | NS | NM_005633 | 2 | 39 222 476 | P1045R | G | C | 0.059 | 0.294 | 0.346 | 0.729 | 0.357 | / | 0 | 2 | VUS | OP1, OP4 | (19) |
| *SOS1* | NS | NM_005633 | 2 | 39 250 139 | Q477R | T | C | 0.133 | 0.521 | 0.476 | 0.233 | 0.341 | / | 0 | 4 | VUS | OM1, OP1, OP4 | (15) |
| *SOS1* | NS | NM_005633 | 2 | 39 249 864 | L569V | G | C | 0.528 | 0.165 | 0.178 | 0.330 | 0.300 | 0.03 | 267 790 | 4 | VUS | OM1, OP1, OP4 | (14) |
| *SOS1* | NS | NM_005633 | 2 | 39 241 107 | P655L* | G | A | 0.173 | 0.162 | 0.702 | 0.091 | 0.282 | 0.8 | 266 630 | 0 | VUS | OM1, OP1, OP4,  SBS1 | (8) |
| *SOS1* | ALL | NM_005633 | 2 | 39 241 981 | N622S | T | C | 0.138 | 0.088 | 0.466 | 0.142 | 0.209 | 0.0008 | 267 062 | 4 | VUS | OM1, OP1, OP4 | (17) |
| *SOS1* | NS | NM_005633 | 2 | 39 214 732 | R1131K | C | T | 0.169 | 0.196 | 0.073 | 0.308 | 0.187 | 0.002 | 225 798 | 2 | VUS | OP1, OP4 | (12) |
| *SOS1* | ALL; NS | NM_005633 | 2 | 39 294 873 | T37A | T | C | 0.118 | 0.060 | 0.282 | 0.229 | 0.172 | 0.01 | 268 248 | 4 | VUS | OM1, OP1, OP4 | (12) (17) |
| *SOS1* | NS | NM_005633 | 2 | 39 213 198 | T1257A | T | C | 0.083 | 0.068 | 0.280 | 0.190 | 0.155 | 0.005 | 267 744 | 2 | VUS | OP1, OP4 | (12) |
| *SOS1* | NS | NM_005633 | 2 | 39 214 706 | L1140I | A | T | 0.109 | 0.079 | 0.240 | 0.190 | 0.155 | 0.003 | 260 862 | 2 | VUS | OP1, OP4 | (12) |
| *SOS1* | pCML | NM_005633 | 2 | 39 281 876 | T200S | G | C | 0.092 | 0.067 | 0.108 | 0.263 | 0.133 | 0.002 | 268 084 | 4 | VUS | OM1, OP1, OP4 | / |
| *SOS1* | pCML | NM_005633 | 2 | 39 240 603 | R722K | C | T | 0.060 | 0.108 | 0.178 | 0.052 | 0.100 | 0.01 | 267 214 | 4 | VUS | OM1, OP1, OP4 | / |
| *SOS1* | NS | NM_005633 | 2 | 39 224 145 | S1000N | C | T | 0.020 | 0.075 | 0.228 | 0.021 | 0.086 | / | 0 | 4 | VUS | OM1, OP1, OP4 | (11) |
| *PTPN11* | RASopathy | NM_002834 | 12 | 112 926 909 | Q510P | A | C | 1.000 | 0.998 | 0.923 | 0.977 | 0.975 | / | 0 | 4 | VUS | OM1, OP1, OP4 | (20) |
| *PTPN11* | RASopathy | NM_002834 | 12 | 112 910 793 | G268C | G | T | 1.000 | 1.000 | 0.858 | 0.962 | 0.955 | 0.0004 | 233 352 | 4 | VUS | OM1, OP1, OP4 | (21) |
| *PTPN11* | RASopathy | NM_002834 | 12 | 112 926 873 | R498L | G | T | 0.993 | 1.000 | 0.856 | 0.941 | 0.948 | / | 0 | 4 | VUS | OM1, OP1, OP4 | (22) |
| *PTPN11* | RASopathy | NM_002834 | 12 | 112 888 195 | F71I | T | A | 0.999 | 1.000 | 0.776 | 0.993 | 0.942 | / | 0 | 6 | LO | OS1, OP1, OP4 | (23) |
| *PTPN11* | RASopathy | NM_002834 | 12 | 112 926 909 | Q510R | A | G | 0.990 | 0.999 | 0.805 | 0.959 | 0.938 | 0.0004 | 236 954 | 4 | VUS | OM1, OP1, OP4 | (24) |
| *PTPN11* | NS/JMML | NM_002834 | 12 | 112 915 455 | F285S | T | C | 0.990 | 1.000 | 0.776 | 0.983 | 0.937 | / | 0 | 5 | VUS | OM1, OP1, OP3,  OP4 | (25) |
| *PTPN11* | RASopathy | NM_002834 | 12 | 112 915 524 | N308T | A | C | 0.950 | 0.979 | 0.865 | 0.941 | 0.934 | / | 0 | 4 | VUS | OM1, OP1, OP4 | (21) |
| *PTPN11* | RASopathy | NM_002834 | 12 | 112 926 269 | T468P | A | C | 1.000 | 0.997 | 0.749 | 0.988 | 0.934 | / | 0 | 4 | VUS | OM1, OP1, OP4 | (26) |
| *PTPN11* | RASopathy | NM_002834 | 12 | 112 888 220 | Q79P | A | C | 0.987 | 0.999 | 0.847 | 0.900 | 0.933 | / | 0 | 4 | VUS | OM1, OP1, OP4 | (27) |
| *PTPN11* | NS/MPD | NM_002834 | 12 | 112 926 872 | R498W | C | T | 0.991 | 1.000 | 0.887 | 0.849 | 0.932 | 0.0004 | 236 952 | 6 | LO | OS1, OP1, OP4 | (28) |
| *PTPN11* | RASopathy | NM_002834 | 12 | 112 888 301 | D106A | A | C | 0.987 | 0.986 | 0.806 | 0.939 | 0.930 | / | 0 | 6 | LO | OS1, OP1, OP4 | (29) |
| *PTPN11* | ALL | NM_002834 | 12 | 112 926 887 | G503R | G | C | 0.993 | 1.000 | 0.732 | 0.992 | 0.929 | 0.0004 | 236 954 | 4 | VUS | OM1, OP1, OP4 | (17) |
| *PTPN11* | NS/ALL | NM_002834 | 12 | 112 926 890 | M504V | A | G | 0.983 | 0.983 | 0.801 | 0.945 | 0.928 | 0.0004 | 236 952 | 6 | LO | OS1, OP1, OP4 | (30) |
| *PTPN11* | RASopathy | NM_002834 | 12 | 112 915 455 | F285C | T | G | 0.921 | 0.999 | 0.844 | 0.944 | 0.927 | / | 0 | 4 | VUS | OM1, OP1, OP4 | (21) |
| *PTPN11* | LS/AML | NM_002834 | 12 | 112 910 827 | Y279C | A | G | 0.950 | 1.000 | 0.775 | 0.973 | 0.925 | / | 0 | 4 | VUS | OM1, OP1, OP4 | (31) |
| *PTPN11* | RASopathy | NM_002834 | 12 | 112 926 270 | T468M | C | T | 0.999 | 0.995 | 0.736 | 0.965 | 0.924 | 0.0004 | 236 648 | 7 | LO | OS1, OP1, OP3, OP4 | (32) |
| *PTPN11* | NS-JMML/MPD | NM_002834 | 12 | 112 926 897 | Q506P | A | C | 0.998 | 0.999 | 0.792 | 0.900 | 0.922 | / | 0 | 4 | VUS | OM1, OP1, OP4 | (33) |
| *PTPN11* | NS/AML | NM_002834 | 12 | 112 888 162 | G60C | G | T | 1.000 | 0.999 | 0.688 | 0.952 | 0.910 | / | 0 | 4 | VUS | OM1, OP1, OP4 | (34) |
| *PTPN11* | RASopathy | NM_002834 | 12 | 112 910 827 | Y279S | A | C | 0.949 | 1.000 | 0.726 | 0.965 | 0.910 | / | 0 | 4 | VUS | OM1, OP1, OP4 | (20) |
| *PTPN11* | NS-JMML/MPD | NM_002834 | 12 | 112 888 195 | F71L | T | C | 0.999 | 1.000 | 0.662 | 0.973 | 0.909 | / | 0 | 4 | VUS | OM1, OP1, OP4 | (33) |
| *PTPN11* | NS-JMML/MPD | NM_002834 | 12 | 112 926 885 | S502L | C | T | 0.984 | 0.999 | 0.672 | 0.976 | 0.908 | / | 0 | 6 | LO | OS3, OP1, OP4 | (33) |
| *PTPN11* | RASopathy | NM_002834 | 12 | 112 884 192 | L43F | C | T | 0.991 | 0.995 | 0.719 | 0.924 | 0.907 | / | 0 | 4 | VUS | OM1, OP1, OP4 | (35) |
| *PTPN11* | ALL | NM_002834 | 12 | 112 888 163 | G60V | G | T | 1.000 | 1.000 | 0.682 | 0.931 | 0.903 | / | 0 | 6 | LO | OS3, OP1, OP4 | (17) |
| *PTPN11* | NS-JMML/MPD | NM_002834 | 12 | 112 888 165 | D61H | G | C | 1.000 | 1.000 | 0.644 | 0.935 | 0.895 | / | 0 | 4 | VUS | OM1, OP1, OP4 | (33) |
| *PTPN11* | RASopathy | NM_002834 | 12 | 112 926 258 | G464A | G | C | 0.999 | 1.000 | 0.582 | 0.985 | 0.892 | / | 0 | 4 | VUS | OM1, OP1, OP4 | (22) |
| *PTPN11* | NS/JMML | NM_002834 | 12 | 112 888 202 | T73I | C | T | 0.999 | 1.000 | 0.589 | 0.954 | 0.886 | / | 0 | 6 | LO | OS1, OP1, OP4 | (36) |
| *PTPN11* | RASopathy | NM_002834 | 12 | 112 926 882 | R501K | G | A | 0.970 | 0.998 | 0.607 | 0.943 | 0.880 | / | 0 | 4 | VUS | OM1, OP1, OP4 | (37) |
| *PTPN11* | NS/JMML | NM_002834 | 12 | 112 926 888 | G503A | G | C | 0.970 | 0.998 | 0.583 | 0.956 | 0.877 | / | 0 | 4 | VUS | OM1, OP1, OP4 | (38) |
| *PTPN11* | NS/ALL | NM_002834 | 12 | 112 888 163 | G60A | G | C | 0.995 | 0.993 | 0.603 | 0.907 | 0.875 | / | 0 | 4 | VUS | OM1, OP1, OP4 | (30) |
| *PTPN11* | RASopathy | NM_002834 | 12 | 112 926 908 | Q510E | C | G | 0.977 | 0.943 | 0.618 | 0.958 | 0.874 | / | 0 | 7 | LO | OS1, OP1, OP3, OP4 | (39) |
| *PTPN11* | NS-JMML/MPD | NM_002834 | 12 | 112 915 456 | F285L | T | G | 0.916 | 1.000 | 0.764 | 0.813 | 0.873 | / | 0 | 4 | VUS | OM1, OP1, OP4 | (33) |
| *PTPN11* | RASopathy | NM_002834 | 12 | 112 910 793 | G268S | G | A | 0.996 | 0.999 | 0.568 | 0.928 | 0.873 | / | 0 | 4 | VUS | OM1, OP1, OP4 | (21) |
| *PTPN11* | RASopathy | NM_002834 | 12 | 112 888 162 | G60S | G | A | 0.990 | 0.989 | 0.583 | 0.890 | 0.863 | / | 0 | 4 | VUS | OM1, OP1, OP4 | (21) |
| *PTPN11* | RASopathy | NM_002834 | 12 | 112 926 248 | A461T | G | A | 0.979 | 0.999 | 0.490 | 0.977 | 0.861 | / | 0 | 5 | VUS | OM1, OP1, OP3,  OP4 | (40) |
| *PTPN11* | NS-JMML/MPD | NM_002834 | 12 | 112 915 523 | N308D | A | G | 0.926 | 0.996 | 0.679 | 0.838 | 0.860 | 0.001 | 236 902 | 7 | LO | OS1, OP1, OP3, OP4 | (36) |
| *PTPN11* | NS-JMML/MPD | NM_002834 | 12 | 112 888 166 | D61G | A | G | 0.990 | 0.999 | 0.499 | 0.920 | 0.852 | / | 0 | 7 | LO | OS1, OP1, OP3, OP4 | (33) |
| *PTPN11* | RASopathy | NM_002834 | 12 | 112 888 168 | Y62N | T | A | 1.000 | 0.978 | 0.483 | 0.900 | 0.840 | 0.0004 | 236 476 | 4 | VUS | OM1, OP1, OP4 | (21) |
| *PTPN11* | ALL | NM_002834 | 12 | 112 915 461 | H287P | A | C | 0.928 | 0.828 | 0.590 | 0.952 | 0.825 | / | 0 | 4 | VUS | OM1, OP1, OP4 | (17) |
| *PTPN11* | RASopathy | NM_002834 | 12 | 112 926 852 | P491L | C | T | 0.935 | 0.943 | 0.741 | 0.675 | 0.824 | / | 0 | 4 | VUS | OM1, OP1, OP4 | (30) |
| *PTPN11* | NS-JMML/MPD | NM_002834 | 12 | 112 888 168 | Y62D | T | G | 0.942 | 0.994 | 0.461 | 0.900 | 0.824 | / | 0 | 4 | VUS | OM1, OP1, OP4 | (33) |
| *PTPN11* | NS/ALL | NM_002834 | 12 | 112 888 220 | Q79R | A | G | 0.976 | 0.964 | 0.424 | 0.933 | 0.824 | / | 0 | 6 | LO | OS1, OP1, OP4 | (10) |
| *PTPN11* | NS-JMML/MPD | NM_002834 | 12 | 112 888 199 | A72G | C | G | 0.971 | 0.993 | 0.401 | 0.924 | 0.822 | / | 0 | 5 | VUS | OM1, OP1, OP3,  OP4 | (33) |
| *PTPN11* | RASopathy | NM_002834 | 12 | 112 888 156 | N58H | A | C | 0.973 | 0.978 | 0.554 | 0.760 | 0.816 | / | 0 | 4 | VUS | OM1, OP1, OP4 | (21) |
| *PTPN11* | RASopathy | NM_002834 | 12 | 112 910 785 | R265Q | G | A | 0.935 | 0.995 | 0.521 | 0.814 | 0.816 | 0.003 | 233 482 | 4 | VUS | OM1, OP1, OP4 | (41) |
| *PTPN11* | NS-JMML/MPD | NM_002834 | 12 | 112 884 189 | T42A | A | G | 0.950 | 0.973 | 0.528 | 0.794 | 0.811 | / | 0 | 4 | VUS | OM1, OP1, OP4 | (30) |
| *PTPN11* | RASopathy | NM_002834 | 12 | 112 888 313 | E110A | G | C | 0.992 | 0.962 | 0.580 | 0.651 | 0.796 | / | 0 | 4 | VUS | OM1, OP1, OP4 | (21) |
| *PTPN11* | RASopathy | NM_002834 | 12 | 112 926 884 | S502T | T | A | 0.887 | 0.985 | 0.365 | 0.942 | 0.795 | / | 0 | 6 | LO | OS1, OP1, OP4 | (42) |
| *PTPN11* | RASopathy | NM_002834 | 12 | 112 888 156 | N58D | A | G | 0.935 | 0.994 | 0.491 | 0.757 | 0.794 | / | 0 | 4 | VUS | OM1, OP1, OP4 | (43) |
| *PTPN11* | NS-JMML/MPD | NM_002834 | 12 | 112 926 884 | S502A | T | G | 0.874 | 0.869 | 0.499 | 0.923 | 0.791 | / | 0 | 6 | LO | OS3, OP1, OP4 | (33) |
| *PTPN11* | RASopathy | NM_002834 | 12 | 112 888 210 | E76Q | G | C | 0.925 | 0.997 | 0.467 | 0.733 | 0.781 | / | 0 | 5 | VUS | OM1, OP1, OP3,  OP4 | (44) |
| *PTPN11* | RASopathy | NM_002834 | 12 | 112 888 158 | N58K | C | A | 0.937 | 0.999 | 0.593 | 0.589 | 0.780 | / | 0 | 4 | VUS | OM1, OP1, OP4 | (45) |
| *PTPN11* | RASopathy | NM_002834 | 12 | 112 888 189 | E69Q | G | C | 0.964 | 0.990 | 0.411 | 0.726 | 0.773 | / | 0 | 4 | VUS | OM1, OP1, OP4 | (45) |
| *PTPN11* | RASopathy | NM_002834 | 12 | 112 910 758 | Q256R | A | G | 0.888 | 0.895 | 0.550 | 0.754 | 0.772 | / | 0 | 4 | VUS | OM1, OP1, OP4 | (45) |
| *PTPN11* | RASopathy | NM_002834 | 12 | 112 888 198 | A72T | G | A | 0.980 | 0.998 | 0.333 | 0.752 | 0.766 | / | 0 | 5 | VUS | OM1, OP1, OP3,  OP4 | (44) |
| *PTPN11* | RASopathy | NM_002834 | 12 | 112 910 776 | L262R | T | G | 0.819 | 0.927 | 0.582 | 0.708 | 0.759 | / | 0 | 4 | VUS | OM1, OP1, OP4 | (41) |
| *PTPN11* | NS/ALL | NM_002834 | 12 | 112 891 083 | E139D | G | C | 0.880 | 0.989 | 0.388 | 0.769 | 0.757 | / | 0 | 6 | LO | OS1, OP1, OP4 | (46) |
| *PTPN11* | RASopathy | NM_002834 | 12 | 112 888 212 | E76D | G | C | 0.883 | 0.998 | 0.421 | 0.703 | 0.751 | / | 0 | 4 | VUS | OM1, OP1, OP4 | (45) |
| *PTPN11* | pCML | NM_002834 | 12 | 112 888 246 | L88I | T | A | 0.755 | 0.965 | 0.601 | 0.555 | 0.719 | / | 0 | 2 | VUS | OM1, OP4, SBP1 | (47) |
| *PTPN11* | NS-JMML/MPD | NM_002834 | 12 | 112 888 165 | D61N | G | A | 0.810 | 0.994 | 0.422 | 0.648 | 0.719 | / | 0 | 7 | LO | OS1, OP1, OP3, OP4 | (33) |
| *PTPN11* | RASopathy | NM_002834 | 12 | 112 888 198 | A72S | G | T | 0.922 | 0.916 | 0.231 | 0.805 | 0.719 | / | 0 | 7 | LO | OS1, OP1, OP3, OP4 | (36) |
| *PTPN11* | RASopathy | NM_002834 | 12 | 112 888 169 | Y62C | A | G | 0.962 | 0.804 | 0.275 | 0.765 | 0.702 | 0.003 | 31 404 | 6 | LO | OS1, OP1, OP4 | (48) |
| *PTPN11* | NS-JMML/MPD | NM_002834 | 12 | 112 915 524 | N308S | A | G | 0.701 | 0.519 | 0.811 | 0.685 | 0.679 | / | 0 | 5 | VUS | OM1, OP1, OP3,  OP4 | (30) |
| *PTPN11* | RASopathy | NM_002834 | 12 | 112 910 835 | I282V | A | G | 0.704 | 0.982 | 0.436 | 0.525 | 0.662 | / | 0 | 6 | LO | OS1, OP1, OP4 | (30) |
| *PTPN11* | RASopathy | NM_002834 | 12 | 112 910 775 | L262F | C | T | 0.763 | 0.935 | 0.411 | 0.529 | 0.660 | / | 0 | 4 | VUS | OM1, OP1, OP4 | (41) |
| *PTPN11* | RASopathy | NM_002834 | 12 | 112 926 851 | P491S | C | T | 0.599 | 0.799 | 0.584 | 0.581 | 0.641 | / | 0 | 6 | LO | OS1, OP1, OP4 | (21) |
| *PTPN11* | NS/ALL | NM_002834 | 12 | 112 888 157 | N58S | A | G | 0.767 | 0.736 | 0.428 | 0.610 | 0.635 | 0.003 | 267 866 | 4 | VUS | OM1, OP1, OP4 | (10) |
| *PTPN11* | RASopathy | NM_002834 | 12 | 112 910 757 | Q256K | C | A | 0.484 | 0.828 | 0.599 | 0.612 | 0.631 | / | 0 | 4 | VUS | OM1, OP1, OP4 | (30) |
| *PTPN11* | RASopathy | NM_002834 | 12 | 112 856 920 | T2I | C | T | 0.414 | 0.934 | 0.212 | 0.213 | 0.443 | / | 0 | 4 | VUS | OM1, OP1, OP4 | (27) |
| *PTPN11* | RASopathy | NM_002834 | 12 | 112 924 286 | T411M | C | T | 0.782 | 0.065 | 0.288 | 0.626 | 0.440 | 0.0009 | 236 420 | 4 | VUS | OM1, OP1, OP4 | (49) |
| *PTPN11* | RASopathy | NM_002834 | 12 | 112 910 772 | L261F | C | T | 0.265 | 0.507 | 0.321 | 0.547 | 0.410 | 0.003 | 31 384 | 4 | VUS | OM1, OP1, OP4 | (41) |
| *PTPN11* | RASopathy | NM_002834 | 12 | 112 910 773 | L261H | T | A | 0.284 | 0.587 | 0.086 | 0.558 | 0.379 | 0.0004 | 233 638 | 4 | VUS | OM1, OP1, OP4 | (41) |
| *PTPN11* | RASopathy | NM_002834 | 12 | 112 915 526 | I309V | A | G | 0.411 | 0.066 | 0.441 | 0.530 | 0.362 | 0.05 | 268 292 | 4 | VUS | OM1, OP1, OP4 | (37) |
| *PTPN11* | RASopathy | NM_002834 | 12 | 112 940 026 | L560F | C | T | 0.251 | 0.126 | 0.160 | 0.206 | 0.186 | 0.003 | 236 564 | 2 | VUS | OP1, OP4 | (27) |
| *PTPN11* | ALL | NM_002834 | 12 | 112 940 006 | T553M | C | T | 0.235 | 0.080 | 0.083 | 0.142 | 0.135 | 0.04 | 267 938 | 0 | VUS | OP4, SBP1 | (17) |
| *NF2* | NF-2 | NM_000268 | 22 | 30 057 219 | L234R | T | G | 1.000 | 0.999 | 0.867 | 0.962 | 0.957 | / | 0 | 4 | VUS | OM1, OP1, OP4 | (50) |
| *NF2* | NF-2 | NM_000268 | 22 | 30 077 457 | L535P | T | C | 1.000 | 0.999 | 0.849 | 0.847 | 0.924 | / | 0 | 6 | LO | OS1, OP1, OP4 | (51) |
| *NF2* | NF-2 | NM_000268 | 22 | 30 032 810 | F62S | T | C | 1.000 | 0.997 | 0.748 | 0.951 | 0.924 | / | 0 | 4 | VUS | OM1, OP1, OP4 | (52) |
| *NF2* | NF-2 | NM_000268 | 22 | 30 067 894 | L360P | T | C | 1.000 | 0.999 | 0.735 | 0.917 | 0.913 | / | 0 | 2 | VUS | OP1, OP4 | (53) |
| *NF2* | NF-2 | NM_000268 | 22 | 30 032 816 | L64P | T | C | 1.000 | 0.999 | 0.710 | 0.929 | 0.910 | / | 0 | 6 | LO | OS1, OP1, OP4 | (54) |
| *NF2* | NF-2 | NM_000268 | 22 | 30 077 451 | K533T | A | C | 0.976 | 0.843 | 0.553 | 0.781 | 0.788 | / | 0 | 2 | VUS | OP1, OP4 | (55) |
| *NF2* | ALL | NM_000268 | 22 | 30 077 492 | E547K | G | A | 0.937 | 0.819 | 0.581 | 0.741 | 0.770 | 0.03 | 268 308 | 2 | VUS | OP1, OP4 | (17) |
| *NF2* | ALL | NM_000268 | 22 | 30 069 399 | E422K | G | A | 0.969 | 0.902 | 0.401 | 0.687 | 0.740 | / | 0 | 2 | VUS | OP1, OP4 | (17) |
| *NF2* | NF-2 | NM_000268 | 22 | 30 054 236 | N220Y | A | T | 1.000 | 0.580 | 0.413 | 0.851 | 0.711 | / | 0 | 4 | VUS | OM1, OP1, OP4 | (56) |
| *NF2* | NF-2 | NM_000268 | 22 | 30 035 155 | E106G | A | G | 0.789 | 0.401 | 0.653 | 0.631 | 0.619 | / | 0 | 6 | LO | OS1, OP1, OP4 | (52) |
| *NF2* | CML | NM_181830 | 22 | 30 060 985 | I190V | A | G | 0.659 | 0.509 | 0.304 | 0.468 | 0.485 | / | 0 | 4 | VUS | OM1, OP1, OP4 | (47) |
| *NF2* | NF-2 | NM_000268 | 22 | 30 032 854 | M77V | A | G | 0.737 | 0.180 | 0.463 | 0.510 | 0.473 | / | 0 | 4 | VUS | OM1, OP1, OP4 | (57) |
| *NF2* | pCML | NM_181831 | 22 | 30 079 034 | I505T | T | C | 0.578 | 0.560 | 0.359 | 0.357 | 0.464 | 0.0004 | 236 948 | 2 | VUS | OP1, OP4 | (47) |
| *NF2* | NF-2 | NM_000268 | 22 | 30 069 372 | K413E | A | G | 0.317 | 0.570 | 0.172 | 0.381 | 0.360 | 0.0004 | 232 320 | 2 | VUS | OP1, OP4 | (51) |
| *NF2* | NF-2 | NM_000268 | 22 | 30 067 870 | T352M | C | T | 0.685 | 0.097 | 0.132 | 0.516 | 0.358 | 0.001 | 236 926 | 2 | VUS | OP1, OP4 | (52) |
| *NF1* | NF-1 | NM_000267 | 17 | 29 496 985 | D186Y | G | T | 1.000 | 0.987 | 0.922 | 0.890 | 0.950 | / | 0 | 2 | VUS | OP1, OP4 | (58) |
| *NF1* | NF-1 | NM_000267 | 17 | 29 553 576 | C709R | T | C | 1.000 | 0.999 | 0.969 | 0.788 | 0.939 | / | 0 | 2 | VUS | OP1, OP4 | (59) |
| *NF1* | NF-1 | NM_001042492 | 17 | 29 663 396 | S2018R | A | C | 0.936 | 1.000 | 0.857 | 0.946 | 0.935 | / | 0 | 2 | VUS | OP1, OP4 | (60) |
| *NF1* | NF-1 | NM_000267 | 17 | 29 556 326 | L898P | T | C | 0.995 | 0.999 | 0.737 | 0.959 | 0.923 | / | 0 | 2 | VUS | OP1, OP4 | (60) |
| *NF1* | NF-1 | NM_000267 | 17 | 29 556 175 | G848R | G | C | 0.981 | 0.999 | 0.967 | 0.740 | 0.922 | / | 0 | 2 | VUS | OP1, OP4 | (61) |
| *NF1* | NF-1 | NM_000267 | 17 | 29 554 565 | W784R | T | C | 0.995 | 1.000 | 0.889 | 0.795 | 0.920 | / | 0 | 2 | VUS | OP1, OP4 | (62) |
| *NF1* | NF-1 | NM_000267 | 17 | 29 556 173 | L847P | T | C | 0.991 | 0.999 | 0.866 | 0.825 | 0.920 | / | 0 | 2 | VUS | OP1, OP4 | (60) |
| *NF1* | NF-1 | NM_000267 | 17 | 29 663 768 | L2067P | T | C | 1.000 | 1.000 | 0.721 | 0.945 | 0.917 | / | 0 | 2 | VUS | OP1, OP4 | (63) |
| *NF1* | NF-1 | NM_000267 | 17 | 29 554 544 | W777G | T | G | 0.995 | 0.997 | 0.756 | 0.901 | 0.912 | / | 0 | 2 | VUS | OP1, OP4 | (60) |
| *NF1* | NF-1 | NM_000267 | 17 | 29 556 392 | L920P | T | C | 0.998 | 0.996 | 0.707 | 0.937 | 0.910 | / | 0 | 4 | VUS | OM1, OP1, OP4 | (64) |
| *NF1* | NF-1 | NM_000267 | 17 | 29 553 535 | L695R | T | G | 1.000 | 0.997 | 0.849 | 0.777 | 0.906 | / | 0 | 2 | VUS | OP1, OP4 | (58) |
| *NF1* | NF-1 | NM_000267 | 17 | 29 586 057 | Q1426P | A | C | 0.936 | 0.996 | 0.707 | 0.966 | 0.901 | / | 0 | 4 | VUS | OM1, OP1, OP4 | (65) |
| *NF1* | NF-1 | NM_000267 | 17 | 29 557 888 | W1048R | T | C | 0.991 | 1.000 | 0.808 | 0.779 | 0.895 | / | 0 | 4 | VUS | OM1, OP1, OP4 | (66) |
| *NF1* | NF-1 | NM_000267 | 17 | 29 586 054 | L1425P | T | C | 0.997 | 1.000 | 0.667 | 0.861 | 0.881 | / | 0 | 4 | VUS | OM1, OP1, OP4 | (62) |
| *NF1* | NF-1 | NM_000267 | 17 | 29 559 129 | L1079P | T | C | 0.993 | 0.999 | 0.702 | 0.813 | 0.877 | / | 0 | 4 | VUS | OM1, OP1, OP4 | (60) |
| *NF1* | NF-1 | NM_000267 | 17 | 29 490 394 | R160T | G | C | 0.949 | 0.987 | 0.689 | 0.843 | 0.867 | / | 0 | 2 | VUS | OP1, OP4 | (60) |
| *NF1* | NF-1 | NM_000267 | 17 | 29 528 062 | L357P | T | C | 0.978 | 0.999 | 0.863 | 0.606 | 0.862 | / | 0 | 4 | VUS | OM1, OP1, OP4 | (67) |
| *NF1* | NF-1 | NM_000267 | 17 | 29 548 946 | S574R | C | A | 0.975 | 1.000 | 0.806 | 0.643 | 0.856 | / | 0 | 2 | VUS | OP1, OP4 | (68) |
| *NF1* | NF-1 | NM_000267 | 17 | 29 490 265 | I117S | T | G | 0.984 | 0.994 | 0.517 | 0.910 | 0.851 | / | 0 | 2 | VUS | OP1, OP4 | (62) |
| *NF1* | NF-1 | NM_000267 | 17 | 29 667 614 | L2317P | T | C | 0.958 | 0.999 | 0.694 | 0.740 | 0.848 | / | 0 | 2 | VUS | OP1, OP4 | (69) |
| *NF1* | NF-1 | NM_000267 | 17 | 29 483 002 | L21R | T | G | 0.981 | 0.994 | 0.706 | 0.698 | 0.845 | / | 0 | 4 | VUS | OM1, OP1, OP4 | (70) |
| *NF1* | NF-1 | NM_000267 | 17 | 29 550 473 | L578R | T | G | 0.997 | 0.992 | 0.689 | 0.680 | 0.840 | / | 0 | 2 | VUS | OP1, OP4 | (58) |
| *NF1* | NF-1 | NM_000267 | 17 | 29 527 519 | A323D | C | A | 0.994 | 0.999 | 0.779 | 0.559 | 0.833 | / | 0 | 2 | VUS | OP1, OP4 | (60) |
| *NF1* | NF-1 | NM_000267 | 17 | 29 587 512 | G1498E | G | A | 1.000 | 0.999 | 0.489 | 0.820 | 0.827 | / | 0 | 2 | VUS | OP1, OP4 | (62) |
| *NF1* | NF-1 | NM_001042492 | 17 | 29 585 423 | R1412T | G | C | 0.998 | 1.000 | 0.398 | 0.900 | 0.824 | / | 0 | 4 | VUS | OM1, OP1, OP4 | (60) |
| *NF1* | NF-1 | NM_000267 | 17 | 29 527 450 | L300P | T | C | 0.984 | 0.986 | 0.596 | 0.705 | 0.818 | / | 0 | 2 | VUS | OP1, OP4 | (58) |
| *NF1* | NF-1 | NM_000267 | 17 | 29 586 056 | Q1426K | C | A | 0.981 | 0.999 | 0.333 | 0.865 | 0.795 | / | 0 | 4 | VUS | OM1, OP1, OP4 | (70) |
| *NF1* | NF-1 | NM_000267 | 17 | 29 585 518 | K1423E | A | G | 0.991 | 1.000 | 0.255 | 0.905 | 0.788 | / | 0 | 4 | VUS | OM1, OP1, OP4 | (62) |
| *NF1* | NF-1 | NM_001042492 | 17 | 29 663 366 | D2008H | G | C | 0.982 | 0.929 | 0.293 | 0.931 | 0.784 | / | 0 | 2 | VUS | OP1, OP4 | (60) |
| *NF1* | NF-1 | NM_000267 | 17 | 29 562 747 | R1276Q | G | A | 0.983 | 0.997 | 0.258 | 0.887 | 0.781 | 0.0004 | 268 062 | 6 | LO | OS1, OP1, OP4 | (58) |
| *NF1* | NF-1 | NM_001042492 | 17 | 29 586 086 | K1457E | A | G | 0.990 | 0.998 | 0.204 | 0.884 | 0.769 | / | 0 | 4 | VUS | OM1, OP1, OP4 | (60) |
| *NF1* | NF-1 | NM_001042492 | 17 | 29 588 770 | A1540E | C | A | 0.994 | 0.964 | 0.327 | 0.784 | 0.767 | / | 0 | 2 | VUS | OP1, OP4 | (60) |
| *NF1* | NF-1 | NM_000267 | 17 | 29 560 133 | R1204W | C | T | 1.000 | 0.997 | 0.301 | 0.759 | 0.764 | / | 0 | 6 | LO | OS1, OP1, OP4 | (62) |
| *NF1* | NF-1 | NM_000267 | 17 | 29 654 518 | V1736D | T | A | 0.994 | 0.999 | 0.328 | 0.721 | 0.761 | / | 0 | 2 | VUS | OP1, OP4 | (70) |
| *NF1* | NF-1 | NM_001042492 | 17 | 29 654 782 | I1845T | T | C | 0.985 | 0.999 | 0.214 | 0.815 | 0.753 | / | 0 | 2 | VUS | OP1, OP4 | (60) |
| *NF1* | CML; NF-1 | NM_000267 | 17 | 29 496 957 | D176E | T | A | 0.962 | 0.944 | 0.437 | 0.641 | 0.746 | 0.4 | 268 052 | 2 | VUS | OP1, OP4 | (47) (60) |
| *NF1* | NF-1 | NM_001042492 | 17 | 29 654 518 | V1757G | T | G | 0.994 | 0.973 | 0.269 | 0.744 | 0.745 | / | 0 | 2 | VUS | OP1, OP4 | (60) |
| *NF1* | NF-1 | NM_000267 | 17 | 29 422 374 | R16P | G | C | 0.935 | 0.997 | 0.694 | 0.321 | 0.737 | / | 0 | 4 | VUS | OM1, OP1, OP4 | (66) |
| *NF1* | NF-1 | NM_000267 | 17 | 29 654 857 | R1849Q | G | A | 0.907 | 0.988 | 0.313 | 0.666 | 0.719 | / | 0 | 7 | LO | OS1, OP1, OP3, OP4 | (59) |
| *NF1* | NF-1 | NM_001042492 | 17 | 29 657 356 | F1884L | T | G | 0.995 | 1.000 | 0.397 | 0.460 | 0.713 | / | 0 | 2 | VUS | OP1, OP4 | (60) |
| *NF1* | NF-1 | NM_000267 | 17 | 29 541 502 | K476E | A | G | 0.896 | 0.961 | 0.387 | 0.436 | 0.670 | / | 0 | 2 | VUS | OP1, OP4 | (60) |
| *NF1* | NF-1 | NM_001042492 | 17 | 29 652 838 | R1612S | G | C | 0.992 | 0.982 | 0.240 | 0.467 | 0.670 | / | 0 | 4 | VUS | OM1, OP1, OP4 | (60) |
| *NF1* | NF-1 | NM_001042492 | 17 | 29 585 519 | K1444R | A | G | 0.946 | 0.764 | 0.096 | 0.849 | 0.664 | / | 0 | 4 | VUS | OM1, OP1, OP4 | (59) |
| *NF1* | NF-1 | NM_000267 | 17 | 29 585 519 | K1423R | A | G | 0.946 | 0.764 | 0.096 | 0.849 | 0.664 | / | 0 | 4 | VUS | OM1, OP1, OP4 | (65) |
| *NF1* | NF-1 | NM_000267 | 17 | 29 560 020 | G1166V | G | T | 0.933 | 0.718 | 0.414 | 0.559 | 0.656 | / | 0 | 2 | VUS | OP1, OP4 | (59) |
| *NF1* | NF-1 | NM_000267 | 17 | 29 560 089 | Q1189R | A | G | 0.984 | 0.829 | 0.235 | 0.571 | 0.655 | 0.0004 | 236 816 | 2 | VUS | OP1, OP4 | (66) |
| *NF1* | NF-1 | NM_000267 | 17 | 29 541 542 | Y489C | A | G | 0.896 | 0.119 | 0.634 | 0.402 | 0.513 | 0.0009 | 236 140 | 6 | LO | OS1, OP1, OP4 | (60) |
| *NF1* | AML | NM_000267 | 17 | 29 685 584 | Q2665R | A | G | 0.841 | 0.346 | 0.279 | 0.442 | 0.477 | / | 0 | 2 | VUS | OP1, OP4 | (17) |
| *NF1* | NF-1 | NM_000267 | 17 | 29 527 563 | D338N | G | A | 0.761 | 0.566 | 0.369 | 0.200 | 0.474 | / | 0 | 2 | VUS | OP1, OP4 | (68) |
| *NF1* | NF-1 | NM_000267 | 17 | 29 664 855 | P2200A | C | G | 0.970 | 0.190 | 0.334 | 0.402 | 0.474 | / | 0 | 2 | VUS | OP1, OP4 | (64) |
| *NF1* | NF-1 | NM_000267 | 17 | 29 422 328 | M1V | A | G | 0.751 | 0.245 | 0.293 | 0.370 | 0.415 | / | 0 | 12 | Onco-genic | OVS1, OM1, OP1,  OP4 | (60) |
| *NF1* | NF-1 | NM_001042492 | 17 | 29 587 421 | S1489G | A | G | 0.652 | 0.071 | 0.223 | 0.549 | 0.374 | / | 0 | 2 | VUS | OP1, OP4 | (60) |
| *NF1* | NF-1 | NM_000267 | 17 | 29 552 152 | G629R | G | A | 0.572 | 0.268 | 0.188 | 0.369 | 0.349 | / | 236 672 | 6 | LO | OS1, OP1, OP4 | (59) |
| *NF1* | NF-1 | NM_000267 | 17 | 29 576 093 | E1356K | G | A | 0.434 | 0.183 | 0.259 | 0.509 | 0.346 | / | 0 | 4 | VUS | OM1, OP1, OP4 | (58) |
| *NF1* | ALL | NM_000267 | 17 | 29 667 607 | A2315S | G | T | 0.754 | 0.139 | 0.245 | 0.241 | 0.345 | / | 0 | 2 | VUS | OP1, OP4 | (17) |
| *NF1* | pCML | ENST00000358273 | 17 | 29 664 529 | S2191P | T | C | 0.396 | 0.174 | 0.363 | 0.350 | 0.321 | 0.0004 | 236 896 | 2 | VUS | OP1, OP4 | / |
| *NF1* | ALL | NM_000267 | 17 | 29 661 949 | Q1948L | A | T | 0.567 | 0.060 | 0.251 | 0.287 | 0.291 | / | 0 | 2 | VUS | OP1, OP4 | (17) |
| *NF1* | CML | NM_000267 | 17 | 29 576 051 | M1342V | A | G | 0.395 | 0.105 | 0.341 | 0.318 | 0.290 | / | 0 | 4 | VUS | OM1, OP1, OP4 | (47) |
| *NF1* | NF-1 | NM_000267 | 17 | 29 683 520 | A2532V | C | T | 0.567 | 0.132 | 0.131 | 0.264 | 0.274 | 0.07 | 267 918 | 2 | VUS | OP1, OP4 | (60) |
| *NF1* | NF-1 | ENST00000356175 | 17 | 29 684 308 | T2610A | A | G | 0.490 | 0.069 | 0.225 | 0.279 | 0.266 | / | 0 | 2 | VUS | OP1, OP4 | (71) |
| *NF1* | NF-1 | NM_001042492 | 17 | 29 665 823 | K2307N | G | T | 0.398 | 0.274 | 0.195 | 0.097 | 0.241 | / | 0 | 2 | VUS | OP1, OP4 | (60) |
| *NF1* | NF-1 | NM_001042492 | 17 | 29 665 722 | A2274S | G | T | 0.392 | 0.174 | 0.201 | 0.058 | 0.206 | / | 0 | 2 | VUS | OP1, OP4 | (60) |
| *NF1* | NF-1 | NM_001042492 | 17 | 29 653 022 | V1674I | G | A | 0.390 | 0.082 | 0.094 | 0.196 | 0.191 | / | 0 | 4 | VUS | OM1, OP1, OP4 | (60) |
| *NF1* | NF-1 | NM_000267 | 17 | 29 552 113 | Q616E | C | G | 0.271 | 0.092 | 0.083 | 0.142 | 0.147 | / | 0 | 2 | VUS | OP1, OP4 | (66) |
| *NF1* | pCML | ENST00000358273 | 17 | 29 592 335 | I1605V | A | G | 0.171 | 0.049 | 0.082 | 0.236 | 0.135 | 0.003 | 31 368 | 4 | VUS | OM1, OP1, OP4 | / |
| *NF1* | NF-1 | NM_000267 | 17 | 29 556 218 | T862S | C | G | 0.080 | 0.094 | 0.132 | 0.080 | 0.097 | 0.003 | 267 750 | 2 | VUS | OP1, OP4 | (60) |
| *NF1* | NF-1 | NM_000267 | 17 | 29 550 488 | K583R | A | G | 0.090 | 0.086 | 0.086 | 0.026 | 0.072 | / | 0 | 2 | VUS | OP1, OP4 | (60) |
| *NF1* | ALL | NM_000267 | 17 | 29 685 568 | I2660V | A | G | 0.030 | 0.068 | 0.101 | 0.042 | 0.060 | / | 0 | 2 | VUS | OP1, OP4 | (17) |
| *NF1* | ALL | NM_000267 | 17 | 29 559 774 | S1124N | G | A | 0.007 | 0.059 | 0.073 | 0.099 | 0.060 | 0.002 | 268 232 | 2 | VUS | OP1, OP4 | (17) |
| *NF1* | pCML | NM_000267 | 17 | 29 552 167 | I634V | A | G | 0.012 | 0.072 | 0.050 | 0.021 | 0.039 | 0.002 | 236 710 | 2 | VUS | OP1, OP4 | (72) |

AF=allele frequency; VUS=variant of uncertain significance; LO=likely oncogenic; NS=Noonan syndrome; ALL=acute lymphoid leukemia; CML=chronic myeloid leukemia; BP=blast phase; AML=acute myeloid leukemia; CP=chronic phase; MPD=myeloproliferative disease; JMML=juvenile myelomonocytic leukemia; LS=LEOPARD syndrome.

* Inherited from unaffected parents

Legend of predicted scores of *in silico* tools:

| **HePPy (2) and REVEL (5)** | | **AlphaMissense (3)** | | **VIPUR (4)** | |
| --- | --- | --- | --- | --- | --- |
| >0.75 | probably pathogenic | >0.564 | likely pathogenic | >0.5 | deleterious |
| 0.5-0.75 | possibly pathogenic | 0.340 - 0.564 | ambigous |  |  |
| <0.5 | probably benign | ≤0.339 | likely benign | <0.5 | neutral |

1. Karczewski KJ, Francioli LC, Tiao G, Cummings BB, Alföldi J, Wang Q, et al. The mutational constraint spectrum quantified from variation in 141,456 humans. Nature. 2020;581(7809):434-43.

2. Hutter S, Baer C, Walter W, Kern W, Haferlach C, Haferlach T. A Novel Machine Learning Based in silico Pathogenicity Predictor for Missense Variants in a Hematological Setting. Blood. 2019;134(Supplement_1):2090-.

3. Cheng J, Novati G, Pan J, Bycroft C, Žemgulytė A, Applebaum T, et al. Accurate proteome-wide missense variant effect prediction with AlphaMissense. Science. 2023;381(6664):eadg7492.

4. Baugh EH, Simmons-Edler R, Müller CL, Alford RF, Volfovsky N, Lash AE, et al. Robust classification of protein variation using structural modelling and large-scale data integration. Nucleic Acids Res. 2016;44(6):2501-13.

5. Ioannidis NM, Rothstein JH, Pejaver V, Middha S, McDonnell SK, Baheti S, et al. REVEL: An Ensemble Method for Predicting the Pathogenicity of Rare Missense Variants. The American Journal of Human Genetics. 2016;99(4):877-85.

6. Carta MG, Tögel L, Hölsken A, Schubart C, Sticht H, Stöhr R, et al. Oncogenicity Variant Interpreter (OncoVI): oncogenicity guidelines implementation to support somatic variants interpretation in precision oncology. The Journal of Molecular Diagnostics. 2026.

7. Tartaglia M, Pennacchio LA, Zhao C, Yadav KK, Fodale V, Sarkozy A, et al. Gain-of-function SOS1 mutations cause a distinctive form of Noonan syndrome. Nat Genet. 2007;39(1):75-9.

8. Roberts AE, Araki T, Swanson KD, Montgomery KT, Schiripo TA, Joshi VA, et al. Germline gain-of-function mutations in SOS1 cause Noonan syndrome. Nature Genetics. 2007;39(1):70-4.

9. Carcavilla A, Cambra A, Santomé JL, Seidel V, Cruz J, Alonso M, et al. Genotypic Findings in Noonan and Non-Noonan RASopathies and Patient Eligibility for Growth Hormone Treatment. J Clin Med. 2023;12(15).

10. Cavé H, Caye A, Strullu M, Aladjidi N, Vignal C, Ferster A, et al. Acute lymphoblastic leukemia in the context of RASopathies. Eur J Med Genet. 2016;59(3):173-8.

11. Zenker M, Horn D, Wieczorek D, Allanson J, Pauli S, van der Burgt I, et al. SOS1 is the second most common Noonan gene but plays no major role in cardio-facio-cutaneous syndrome. J Med Genet. 2007;44(10):651-6.

12. Lepri F, De Luca A, Stella L, Rossi C, Baldassarre G, Pantaleoni F, et al. SOS1 mutations in Noonan syndrome: molecular spectrum, structural insights on pathogenic effects, and genotype-phenotype correlations. Hum Mutat. 2011;32(7):760-72.

13. Beneteau C, Cavé H, Moncla A, Dorison N, Munnich A, Verloes A, et al. SOS1 and PTPN11 mutations in five cases of Noonan syndrome with multiple giant cell lesions. Eur J Hum Genet. 2009;17(10):1216-21.

14. Denayer E, Devriendt K, de Ravel T, Van Buggenhout G, Smeets E, Francois I, et al. Tumor spectrum in children with Noonan syndrome and SOS1 or RAF1 mutations. Genes Chromosomes Cancer. 2010;49(3):242-52.

15. Longoni M, Moncini S, Cisternino M, Morella IM, Ferraiuolo S, Russo S, et al. Noonan syndrome associated with both a new Jnk-activating familial SOS1 and a de novo RAF1 mutations. Am J Med Genet A. 2010;152a(9):2176-84.

16. Jongmans MC, Hoogerbrugge PM, Hilkens L, Flucke U, van der Burgt I, Noordam K, et al. Noonan syndrome, the SOS1 gene and embryonal rhabdomyosarcoma. Genes Chromosomes Cancer. 2010;49(7):635-41.

17. Zhang J, Walsh MF, Wu G, Edmonson MN, Gruber TA, Easton J, et al. Germline Mutations in Predisposition Genes in Pediatric Cancer. N Engl J Med. 2015;373(24):2336-46.

18. Baban A, Olivini N, Lepri FR, Calì F, Mucciolo M, Digilio MC, et al. SOS1 mutations in Noonan syndrome: Cardiomyopathies and not only congenital heart defects! Report of six patients including two novel variants and literature review. Am J Med Genet A. 2019;179(10):2083-90.

19. van Trier DC, Rinne T, Noordam K, Draaisma JM, van der Burgt I. Variable phenotypic expression in a large Noonan syndrome family segregating a novel SOS1 mutation. Am J Med Genet A. 2017;173(11):2968-72.

20. Keren B, Hadchouel A, Saba S, Sznajer Y, Bonneau D, Leheup B, et al. PTPN11 mutations in patients with LEOPARD syndrome: a French multicentric experience. J Med Genet. 2004;41(11):e117.

21. Tartaglia M, Martinelli S, Stella L, Bocchinfuso G, Flex E, Cordeddu V, et al. Diversity and functional consequences of germline and somatic PTPN11 mutations in human disease. Am J Hum Genet. 2006;78(2):279-90.

22. Sarkozy A, Conti E, Digilio MC, Marino B, Morini E, Pacileo G, et al. Clinical and molecular analysis of 30 patients with multiple lentigines LEOPARD syndrome. J Med Genet. 2004;41(5):e68.

23. Niihori T, Aoki Y, Ohashi H, Kurosawa K, Kondoh T, Ishikiriyama S, et al. Functional analysis of PTPN11/SHP-2 mutants identified in Noonan syndrome and childhood leukemia. J Hum Genet. 2005;50(4):192-202.

24. Bertola DR, Pereira AC, Passetti F, de Oliveira PSL, Messiaen L, Gelb BD, et al. Neurofibromatosis–Noonan syndrome: Molecular evidence of the concurrence of both disorders in a patient. American Journal of Medical Genetics Part A. 2005;136A(3):242-5.

25. Prontera P, Pantaleoni F, Martinelli S, Mastrodicasa E, Stangoni G, Barboni G, et al. Germline PTPN11 mutation affecting exon 8 in a case of syndromic juvenile myelomonocytic leukemia. Leukemia Research. 2011;35(3):e13-e4.

26. Seishima M, Mizutani Y, Shibuya Y, Arakawa C, Yoshida R, Ogata T. Malignant melanoma in a woman with LEOPARD syndrome: identification of a germline PTPN11 mutation and a somatic BRAF mutation. British Journal of Dermatology. 2007;157(6):1297-9.

27. Sarkozy A, Conti E, Seripa D, Digilio MC, Grifone N, Tandoi C, et al. Correlation between PTPN11 gene mutations and congenital heart defects in Noonan and LEOPARD syndromes. J Med Genet. 2003;40(9):704-8.

28. Kratz CP, Niemeyer CM, Castleberry RP, Cetin M, Bergsträsser E, Emanuel PD, et al. The mutational spectrum of PTPN11 in juvenile myelomonocytic leukemia and Noonan syndrome/myeloproliferative disease. Blood. 2005;106(6):2183-5.

29. Jafarov T, Ferimazova N, Reichenberger E. Noonan-like syndrome mutations in PTPN11 in patients diagnosed with cherubism. Clin Genet. 2005;68(2):190-1.

30. Binder G, Neuer K, Ranke MB, Wittekindt NE. PTPN11 mutations are associated with mild growth hormone resistance in individuals with Noonan syndrome. J Clin Endocrinol Metab. 2005;90(9):5377-81.

31. Uçar C, Calýskan U, Martini S, Heinritz W. Acute myelomonocytic leukemia in a boy with LEOPARD syndrome (PTPN11 gene mutation positive). J Pediatr Hematol Oncol. 2006;28(3):123-5.

32. Digilio MC, Conti E, Sarkozy A, Mingarelli R, Dottorini T, Marino B, et al. Grouping of multiple-lentigines/LEOPARD and Noonan syndromes on the PTPN11 gene. Am J Hum Genet. 2002;71(2):389-94.

33. Strullu M, Caye A, Lachenaud J, Cassinat B, Gazal S, Fenneteau O, et al. Juvenile myelomonocytic leukaemia and Noonan syndrome. Journal of Medical Genetics. 2014;51(10):689-97.

34. La Starza R, Rosati R, Roti G, Gorello P, Bardi A, Crescenzi B, et al. A new NDE1/PDGFRB fusion transcript underlying chronic myelomonocytic leukaemia in Noonan Syndrome. Leukemia. 2007;21(4):830-3.

35. Weismann CG, Hager A, Kaemmerer H, Maslen CL, Morris CD, Schranz D, et al. PTPN11 mutations play a minor role in isolated congenital heart disease. American Journal of Medical Genetics Part A. 2005;136A(2):146-51.

36. Kosaki K, Suzuki T, Muroya K, Hasegawa T, Sato S, Matsuo N, et al. PTPN11 (Protein-Tyrosine Phosphatase, Nonreceptor-Type 11) Mutations in Seven Japanese Patients with Noonan Syndrome. The Journal of Clinical Endocrinology & Metabolism. 2002;87(8):3529-33.

37. Tartaglia M, Kalidas K, Shaw A, Song X, Musat DL, van der Burgt I, et al. PTPN11 mutations in Noonan syndrome: molecular spectrum, genotype-phenotype correlation, and phenotypic heterogeneity. Am J Hum Genet. 2002;70(6):1555-63.

38. Jongmans MCJ, van der Burgt I, Hoogerbrugge PM, Noordam K, Yntema HG, Nillesen WM, et al. Cancer risk in patients with Noonan syndrome carrying a PTPN11 mutation. European Journal of Human Genetics. 2011;19(8):870-4.

39. Takahashi K, Kogaki S, Kurotobi S, Nasuno S, Ohta M, Okabe H, et al. A novel mutation in the PTPN11 gene in a patient with Noonan syndrome and rapidly progressive hypertrophic cardiomyopathy. Eur J Pediatr. 2005;164(8):497-500.

40. Yoshida R, Nagai T, Hasegawa T, Kinoshita E, Tanaka T, Ogata T. Two novel and one recurrent PTPN11 mutations in LEOPARD syndrome. American Journal of Medical Genetics Part A. 2004;130A(4):432-4.

41. Pannone L, Bocchinfuso G, Flex E, Rossi C, Baldassarre G, Lissewski C, et al. Structural, Functional, and Clinical Characterization of a Novel PTPN11 Mutation Cluster Underlying Noonan Syndrome. Human Mutation. 2017;38(4):451-9.

42. Kondoh T, Ishii E, Aoki Y, Shimizu T, Zaitsu M, Matsubara Y, et al. Noonan syndrome with leukaemoid reaction and overproduction of catecholamines: a case report. European Journal of Pediatrics. 2003;162(7):548-9.

43. Ferreira LV, Souza SA, Arnhold IJ, Mendonca BB, Jorge AA. PTPN11 (protein tyrosine phosphatase, nonreceptor type 11) mutations and response to growth hormone therapy in children with Noonan syndrome. J Clin Endocrinol Metab. 2005;90(9):5156-60.

44. Mason-Suares H, Toledo D, Gekas J, Lafferty KA, Meeks N, Pacheco MC, et al. Juvenile myelomonocytic leukemia-associated variants are associated with neo-natal lethal Noonan syndrome. Eur J Hum Genet. 2017;25(4):509-11.

45. Musante L, Kehl HG, Majewski F, Meinecke P, Schweiger S, Gillessen-Kaesbach G, et al. Spectrum of mutations in PTPN11 and genotype-phenotype correlation in 96 patients with Noonan syndrome and five patients with cardio-facio-cutaneous syndrome. Eur J Hum Genet. 2003;11(2):201-6.

46. Karow A, Steinemann D, Göhring G, Hasle H, Greiner J, Harila-Saari A, et al. Clonal duplication of a germline PTPN11 mutation due to acquired uniparental disomy in acute lymphoblastic leukemia blasts from a patient with Noonan syndrome. Leukemia. 2007;21(6):1303-5.

47. Krumbholz M, Dolnik A, Sträng E, Ghete T, Skambraks S, Hutter S, et al. A high proportion of germline variants in pediatric chronic myeloid leukemia. Mol Cancer. 2024;23(1):206.

48. Tartaglia M, Mehler EL, Goldberg R, Zampino G, Brunner HG, Kremer H, et al. Mutations in PTPN11, encoding the protein tyrosine phosphatase SHP-2, cause Noonan syndrome. Nature Genetics. 2001;29(4):465-8.

49. Bertola DR, Pereira AC, de Oliveira PS, Kim CA, Krieger JE. Clinical variability in a Noonan syndrome family with a new PTPN11 gene mutation. Am J Med Genet A. 2004;130a(4):378-83.

50. Baser ME, Kluwe L, Mautner VF. Germ-line NF2 mutations and disease severity in neurofibromatosis type 2 patients with retinal abnormalities. Am J Hum Genet. 1999;64(4):1230-3.

51. Bourn D, Evans G, Mason S, Tekes S, Trueman L, Strachan T. Eleven novel mutations in the NF2 tumour suppressor gene. Hum Genet. 1995;95(5):572-4.

52. Bourn D, Carter SA, Mason S, Gareth D, Evans R, Strachan T. Germline mutations in the neurofibromatosis type 2 tumour suppressor gene. Hum Mol Genet. 1994;3(5):813-6.

53. Mérel P, Hoang-Xuan K, Sanson M, Bijlsma E, Rouleau G, Laurent-Puig P, et al. Screening for germ-line mutations in the NF2 Gene. Genes, Chromosomes and Cancer. 1995;12(2):117-27.

54. Demange L, de Moncuit C, Thomas G, Olschwang S. Analyse phénotypique de 154 patients porteurs d’une mutation constitutionnelle du gène NF2. Revue Neurologique. 2007;163(11):1031-8.

55. Hung G, Faudoa R, Baser ME, Xue Z, Kluwe L, Slattery W, et al. Neurofibromatosis 2 phenotypes and germ-line NF2 mutations determined by an RNA mismatch method and loss of heterozygosity analysis in NF2 schwannomas. Cancer Genet Cytogenet. 2000;118(2):167-8.

56. Ruttledge MH, Andermann AA, Phelan CM, Claudio JO, Han FY, Chretien N, et al. Type of mutation in the neurofibromatosis type 2 gene (NF2) frequently determines severity of disease. Am J Hum Genet. 1996;59(2):331-42.

57. Evans DG, Trueman L, Wallace A, Collins S, Strachan T. Genotype/phenotype correlations in type 2 neurofibromatosis (NF2): evidence for more severe disease associated with truncating mutations. J Med Genet. 1998;35(6):450-5.

58. Upadhyaya M, Spurlock G, Kluwe L, Chuzhanova N, Bennett E, Thomas N, et al. The spectrum of somatic and germline NF1 mutations in NF1 patients with spinal neurofibromas. neurogenetics. 2009;10(3):251-63.

59. Anastasaki C, Morris SM, Gao F, Gutmann DH. Children with 5'-end NF1 gene mutations are more likely to have glioma. Neurol Genet. 2017;3(5):e192.

60. Tsipi M, Poulou M, Fylaktou I, Kosma K, Tsoutsou E, Pons M-R, et al. Phenotypic expression of a spectrum of Neurofibromatosis Type 1 (NF1) mutations identified through NGS and MLPA. Journal of the Neurological Sciences. 2018;395:95-105.

61. Pascual-Castroviejo I, Pascual-Pascual SI, Velazquez-Fragua R, Botella P, Viaño J. Familial spinal neurofibromatosis. Neuropediatrics. 2007;38(2):105-8.

62. Ars E, Kruyer H, Morell M, Pros E, Serra E, Ravella A, et al. Recurrent mutations in the NF1 gene are common among neurofibromatosis type 1 patients. J Med Genet. 2003;40(6):e82.

63. Kaufmann D, Müller R, Bartelt B, Wolf M, Kunzi-Rapp K, Hanemann CO, et al. Spinal neurofibromatosis without café-au-lait macules in two families with null mutations of the NF1 gene. Am J Hum Genet. 2001;69(6):1395-400.

64. Kluwe L, Tatagiba M, Fünsterer C, Mautner VF. NF1 mutations and clinical spectrum in patients with spinal neurofibromas. J Med Genet. 2003;40(5):368-71.

65. Hutter S, Piro RM, Waszak SM, Kehrer-Sawatzki H, Friedrich RE, Lassaletta A, et al. No correlation between NF1 mutation position and risk of optic pathway glioma in 77 unrelated NF1 patients. Hum Genet. 2016;135(5):469-75.

66. Bolcekova A, Nemethova M, Zatkova A, Hlinkova K, Pozgayova S, Hlavata A, et al. Clustering of mutations in the 5' tertile of the NF1 gene in Slovakia patients with optic pathway glioma. Neoplasma. 2013;60(6):655-65.

67. Messiaen L, Riccardi V, Peltonen J, Maertens O, Callens T, Karvonen SL, et al. Independent <em>NF1</em> mutations in two large families with spinal neurofibromatosis. Journal of Medical Genetics. 2003;40(2):122-6.

68. Sharif S, Upadhyaya M, Ferner R, Majounie E, Shenton A, Baser M, et al. A molecular analysis of individuals with neurofibromatosis type 1 (NF1) and optic pathway gliomas (OPGs), and an assessment of genotype-phenotype correlations. J Med Genet. 2011;48(4):256-60.

69. Wu R, López-Correa C, Rutkowski JL, Baumbach LL, Glover TW, Legius E. Germline mutations in NF1 patients with malignancies. Genes Chromosomes Cancer. 1999;26(4):376-80.

70. Bottillo I, Ahlquist T, Brekke H, Danielsen SA, van den Berg E, Mertens F, et al. Germline and somatic NF1 mutations in sporadic and NF1-associated malignant peripheral nerve sheath tumours. The Journal of Pathology. 2009;217(5):693-701.

71. Upadhyaya M, Maynard J, Osborn M, Huson SM, Ponder M, Ponder BA, et al. Characterisation of germline mutations in the neurofibromatosis type 1 (NF1) gene. J Med Genet. 1995;32(9):706-10.

72. Krivokuca A, Mihajlovic M, Susnjar S, Spasojevic IB, Minic I, Popovic L, et al. Mutational profile of hereditary breast and ovarian cancer – Establishing genetic testing guidelines in a developing country. Current Problems in Cancer. 2022;46(1):100767.
